# Development of Natural Colorant-Loaded Oleogels and Their Use in Cookies

**DOI:** 10.3390/foods15173011

**Published:** 2026-08-26

**Authors:** Emine Bakir, Hasan Yalcin

**Affiliations:** 1 Food Processing Department, Armutlu Vocational School, Yalova University, Yalova 77500, Türkiye; 2 Food Engineering Department, Graduate School of Natural and Applied Science, Erciyes University, Kayseri 38039, Türkiye; 3 Food Engineering Department, Engineering Faculty, Erciyes University, Kayseri 38030, Türkiye; hyalcin@erciyes.edu.tr

**Keywords:** oleogel, natural colorant, cookie, tomato, carrot, spinach, color

## Abstract

This study aimed to investigate the production of color-enriched oleogels using sunflower oil enriched with natural pigments, and to evaluate their potential as fat replacers in cookie formulations. Tomato, carrot, and spinach were used as natural color sources to obtain red, orange, and green colored oils, which were subsequently structured with candelilla wax to produce colored oleogels. The physicochemical, structural, oxidative, textural, and color properties of both oils and oleogels were evaluated. Oleogel’s performance was further assessed in cookie systems compared with margarine-based control formulations. The results showed that all oleogels exhibited high oil-binding capacity (99.44–99.84%) and stable gelation behavior. Oxidative stability analysis indicated a tendency for peroxide, free fatty acid, and p-anisinide values to increase during storage. FTIR analysis confirmed that no chemical modification occurred in the lipid structure, indicating that oleogel formation was governed by physical interactions. Color analysis demonstrated that pigment incorporation significantly affected L*, a*, and b* values, while spinach oleogel showed superior color stability over 90 days. Oleogel-based cookies generally exhibited lower hardness and higher spread ratios (4.71–5.38) than the margarine control (3.04), while their expansion ratios (137.50–162.50%) were lower than that of the control (212.50%); however, baking weight loss remained unaffected. This study demonstrates that naturally pigment-enriched oleogels can serve as healthy fat replacers within clean-label bakery product development.

## 1. Introduction

In recent years, increasing consumer awareness has led to greater recognition of the adverse health effects associated with the excessive consumption of saturated and trans fatty acids. However, reducing the levels of these fats in food formulations remains a significant challenge, as fats play essential structural, sensory, and technological roles in food systems [1]. Consequently, there has been growing interest in developing healthier fat alternatives for food applications. In response to public health concerns, many countries have implemented regulatory measures to improve the nutritional quality of foods by reducing the content of components such as trans fats and saturated fatty acids [2]. Since excessive intake of saturated and trans fats has been associated with an increased risk of cardiovascular diseases, Type 2 diabetes, and hypercholesterolemia, the World Health Organization has recommended that their consumption should not exceed 10% of total daily energy intake [3]. These concerns have stimulated extensive research into the development of novel solid-fat alternatives. Among the emerging approaches, oleogels, structured lipid systems that convert liquid oils into semi-solid materials, have attracted considerable attention as promising substitutes for conventional solid fats, which are generally regarded as less desirable from a nutritional standpoint.

Oleogels are structured lipid systems in which liquid oil is immobilized within a three-dimensional network formed through non-covalent interactions. The gelation process is achieved by structuring agents, known as oleogelators, which promote the formation of a solid-like network through interactions such as hydrogen bonding, electrostatic forces, and van der Waals interactions, thereby entrapping the liquid oil phase [4]. A wide range of compounds have been investigated as oleogelators, including natural waxes, mono- and diglycerides, fatty alcohols and esters, phospholipids, and phytosterols. Among these, wax-based oleogelators have attracted particular attention due to their superior structuring ability, capacity to improve textural properties, and high oil-binding efficiency. Furthermore, their ability to form stable gel networks at relatively low concentrations makes them highly effective oleogelators for food applications [5].

Candelilla wax is considered one of the most efficient wax-based oleogelators used in oleogelation systems. It is primarily composed of 27–35% wax esters, 7–10% free fatty acids, 10–15% free fatty alcohols, and 50–65% hydrocarbons. Candelilla wax is obtained from the leaves of *Euphorbia cerifera* and *Euphorbia antisyphilitica*, plant species native to northern Mexico and the southwestern United States [2,5]. Owing to its unique chemical composition and strong gel-forming ability, candelilla wax has been extensively utilized in the development of oleogel-based food systems as a healthier alternative to conventional solid fats.

In addition to improving the fatty acid profile of food products, the nutritional value of oleogels can be further enhanced by incorporating bioactive compounds [6]. Oleogels have also been employed as delivery systems to encapsulate and transport functional ingredients such as antioxidants and flavor compounds, offering advantages including improved stability and controlled release properties [7]. This functionality may contribute not only to enhanced nutritional quality but also to improved product stability and extended shelf life, thereby increasing overall product attractiveness [6].

Natural pigments are among the active compounds that may influence the structural properties of wax-based oleogels [8]. Chlorophyll is a lipophilic pigment responsible for the green color of olive oil, while carotenoids represent another class of lipophilic compounds with high antioxidant capacity [9]. Lycopene, a red carotenoid abundant in tomatoes, has attracted considerable attention for its role in free radical scavenging, prevention of chronic diseases, and immune system support. However, like other carotenoids, lycopene is highly susceptible to degradation by environmental factors such as heat and light due to its unsaturated, lipophilic structure, which can lead not only to a loss of its beneficial biological activity but also to color deterioration [10].

Oleogels can protect bioactive compounds against degradation, enable controlled release, and enhance their bioavailability, making them suitable systems for functional food applications. As a promising class of structured edible lipids with unique physicochemical properties and versatile applications in the food industry, oleogels have been utilized in confectionery, bakery products, margarines, and meat products due to their ability to improve texture, reduce saturated fat content, and provide clean-label alternatives [7].

Cookies are widely consumed bakery products that are popular across diverse consumer groups. In bakery applications, particularly in cookie formulations, butter and margarine are among the most commonly used fat sources, as they provide a versatile fat system that contributes to the desired sensory attributes and technological functionality of the final product [11]. Cookies are high-fat products, typically containing approximately 20–30% fat, which is traditionally derived from saturated fat sources [4].

In response to increasing health concerns, there is a growing interest in developing healthier solid fat alternatives with reduced saturated fat content, and oleogels have emerged as a promising solution to meet this demand [12]. While there are some studies on the development of various oleogel systems as alternatives to solid fats in cookie production [2,4,13,14] no studies have been found that examine the use of oleogel systems enriched with natural color pigments in cookie making and the effects of their use on the shelf life of cookies and the stability of natural color pigments.

In the present study, fat-soluble color pigments were extracted from vegetable materials rich in natural color pigments, using sunflower oil as a green solvent. Colored oleogels were produced from sunflower oils colored with vegetable-specific color pigments using candelilla wax. The physicochemical, structural, and functional properties of the colored oils, the resulting oleogels, and the oleogel-containing cookies were comprehensively characterized. Furthermore, quality attributes and storage stability were monitored over a 90-day storage period and compared with those of control samples prepared using conventional fat systems.

Considering the growing consumer demand for healthier, more natural food products, together with the increasing interest in oleogel technology as an innovative strategy for fat replacement, naturally colored oleogels represent a promising approach to developing value-added bakery products. Considering the widespread use of saturated fat-rich solid fats and artificial colorings in pastry products, this study suggests that incorporating oleogels colored with natural pigments into cookie formulations will help reduce dependence on both traditional solid fats and artificial coloring. Therefore, oleogel systems with natural pigments and the cookies produced from them have the potential to serve as innovative consumer-oriented alternatives in the baking industry.

## 2. Materials and Methods

### 2.1. Material

Fresh, ripe tomatoes (*Lycopersicon esculentum*), carrots (*Daucus carota* L.), and spinach (*Spinacia oleracea*) were purchased from local markets and used as natural sources of color pigments in the present study. During selection, care was taken to ensure that the vegetables were free of visible defects or blemishes and exhibited uniform size. All samples were stored at +4 °C until further use.

Candelilla wax (CDW) was obtained from Ravago Petrochemicals (Istanbul, Türkiye). All chemicals used in the study were purchased from Merck (Darmstadt, Germany) and Sigma-Aldrich Chemie GmbH (Darmstadt, Germany) and were of analytical grade purity.

### 2.2. Extraction of Color Pigments

The vegetables used for maceration were dried as described by Engin et al. [15], who reported the highest pigment yield when drying the samples in a hot-air oven at 55 °C for 8 h. In a study investigating the optimization of lycopene extraction from tomato peels via maceration, maximum extraction efficiency (99.3% of total lycopene) was obtained using a biomass-to-oil ratio of 2.5% (*w*/*v*), an extraction temperature of 80 °C, and magnetic stirring at 400 rpm for 45 min [16]. Based on these findings, oil-soluble pigments were extracted from the dried vegetable materials under the reported extraction conditions. The drying and maceration parameters used in these studies were applied separately for all vegetables in our study. Tomatoes, carrots, and spinach, dried at 55 °C for 8 h were subjected to maceration at 80 °C for 45 min with a 2.5% biomass/oil ratio. The oil-soluble color pigments that pass from the vegetables into the oil under the influence of temperature form the source of the colored oil used in oleogel production. Following extraction, the oils were filtered through coarse filter paper to remove residual plant material, yielding pigment-enriched oils suitable for subsequent oleogel preparation. In our study, 3 different colored oil extracts were obtained in addition to the control sample.

- COil: Control oil (Without colorant sunflower oil);
- ROil: Oil obtained by extracting tomato pigments;
- OOil: Oil obtained by extracting carrot pigments;
- GOil: Oil obtained by extracting spinach pigments.

### 2.3. Preparation of Oleogels

For oleogel preparation, candelilla wax was dispersed in sunflower oil and heated in a water bath maintained at 80 °C. Based on preliminary experiments and literature research, the minimum candelilla wax concentration that allowed oleogel formation (2.5%, *w*/*w*) was used. Once the wax had completely melted, the oil–wax mixture was transferred to a magnetic stirrer and mixed at 200 rpm for 3 min at 80 °C to ensure complete homogenization. The resulting mixture was then poured into test tubes and left undisturbed at room temperature for 24 h to allow oleogel formation [17,18]. After gelation, the oleogels were stored at room temperature for 3 months, and color, texture, peroxide value, free fatty acid, and p-anisidin analyses were performed at 15-day intervals throughout the storage period.

In this study, three differently colored oleogels were formulated using sunflower oil enriched with different natural pigments, together with a control oleogel prepared without color addition. The colored oleogels were produced using distinct pigment-enriched oil phases, yielding three separate oleogel systems with different color characteristics. For ease of presentation, the oleogel samples were coded as follows:

- CO: Control Oleogel (Without color);
- RO: Red Oleogel (prepared with tomato-derived color pigment-enriched oil);
- OO: Orange Oleogel (prepared with carrot-derived color pigment-enriched oil);
- GO: Green Oleogel (prepared with spinach-derived color pigment-enriched oil).

These codes were used consistently throughout the physicochemical, structural, and cookie formulation analyses. All oleogels were prepared using the same procedure.

### 2.4. Preparation of Cookies

Cookie samples were prepared with slight modifications to the formulation reported by Pestoric et al. [19]. The oleogels produced in the present study were used as the fat source in the experimental formulations, while margarine was employed as the conventional fat source in the control formulation.

The cookies were prepared using freshly prepared oleogels on day 0, prior to the initiation of the storage period. The cookie formulation consisted of 100 g of wheat flour, 1 g of baking powder, 1 g of salt, 30 g of powdered sugar, 30 g of oleogel, and 25 mL of water. For the control formulation, oleogel was replaced with an equivalent amount of margarine. Cookies were prepared by shaping the dough using a standard mold with a diameter of 60 mm and a height of 8 mm and baked in an oven at 180 °C for 15 min. After baking, the cookies were allowed to cool to room temperature, after which the initial-day analyses were performed. For the storage analyses, the cookies were grouped and packaged together in transparent, thin polyethylene bags, which were hermetically sealed to minimize air exposure. The packaged cookies were stored at room temperature in the dark under ambient relative humidity of an average of 50% throughout the storage period.

### 2.5. Analysis

#### 2.5.1. Oleogels Analysis

Oil-binding capacity

The oil-binding capacity (OBC) of the oleogels was determined according to the method described by Gao et al. [13] with minor modifications: (a) Approximately 1 g of the oleogel sample was transferred into a pre-weighed Eppendorf tube and stored at 4 °C for 1 h to ensure complete gel formation; (b) following gelation, the tubes were weighed again, and the samples were equilibrated at room temperature for 15 min and centrifuged at 10,000 rpm for 15 min using a centrifuge (Hettich, Kirchlengern, Germany); (c) after centrifugation, the released oil was carefully removed, and the tubes containing the remaining gel were reweighed. The analyses were conducted only on day 0 of storage. The oil-binding capacity was calculated using the following equation:

$$\%\;Oil\;release=\frac{\left[(b-a)-(c-a)\right]}{\left(b-a\right)}\times 100$$

% OBC=100−% Oil release

Gelation Time

The gelation time of the oleogel samples was determined according to the method described by Dassanayake et al. [20]. Glass test tubes were filled to approximately half of their volume with oleogel samples and placed in a water bath at 90 °C. The samples were maintained at this temperature for 2 h to ensure complete melting of the oleogels and attainment of isothermal equilibrium. Subsequently, the tubes were removed from the water bath and allowed to cool at room temperature. The time required for gel reformation was recorded as the gelation time. Gel formation was considered complete when no flow was observed upon tilting the test tube to a horizontal position (90° inclination relative to the vertical axis) [20]. The analyses were conducted only on day 0 of storage.

Melting point determination

The softening and melting points of oleogels were measured according to the AOCS official method CC 1–25 at the initial storage time (day 0) only [21]. Glass tubes containing the oleogels were placed in a water bath set to an initial temperature of 38 °C, and the temperature was increased by 1 °C every 3 min. Two temperatures were recorded during the heating process. The first point is the temperature at which the oleogel sample begins to soften and can no longer support a fixed weight, such as a needle on its surface. The second point is the temperature recorded when the oleogel becomes completely clear and liquid (clearing point). The melting point of the oleogels was determined by averaging these two temperatures. The melting point was measured three times for each oleogel.

Color Analysis

The color parameters of the prepared oils and oleogels were determined using a precision colorimeter (FRU, precise color reader, WR-10, Shenzhen, China), and the results were expressed in terms of the CIE L*a*b* color space. In this system, L* indicates lightness ranging from 0 (black) to 100 (white), while a* represents the green (−a*) to red (+a*) axis, and b* represents the blue (−b*) to yellow (+b*) axis [17]. Total color difference (*ΔE*) was calculated by the following equation [11]:

$$\Delta E=\sqrt{{\left(L^{*}-{L0}^{*}\right)}^{2}+{\left(a^{*}-{a0}^{*}\right)}^{2}+{\left(b^{*}-{b0}^{*}\right)}^{2}}$$

where L*, a*, and b* are color parameters of oleogels, while *L*0*, *a*0*, and *b*0* are color parameters of control oleogel.

All samples were analyzed at 15-day intervals over a 90-day storage period to monitor potential changes in color stability.

Texture Analysis

Texture profile analysis of the oleogel samples was performed using a texture analyzer (Brookfield CT3, Middleboro, MA, USA) in compression mode to determine hardness at 15-day intervals throughout the 90-day storage period. Measurements were conducted on matured oleogels (24 h at 25 ± 1 °C) using a conical probe (TA2/1000, Brookfield, Middleboro, MA, USA). A 1 kg load cell was used for all measurements [22].

Texture parameters were automatically recorded using TexturePro CT V1.9 Build 35 software. The test conditions were set as follows: pre-test speed of 2.00 mm s^−1^, test speed of 1.00 mm s^−1^, and post-test speed of 2.00 mm s^−1^ [8].

Peroxide Value

The peroxide value (PV) of the samples was determined using a modified titrimetric method based on [23] and adapted according to the AOAC method, at 15-day intervals throughout the 90-day storage period. Briefly, 5 g of the sample (m) was accurately weighed and transferred into an Erlenmeyer flask. Subsequently, 30 mL of an acetic acid–chloroform mixture (3:2, *v*/*v*) was added, and the flask was vigorously shaken for 1 min to ensure complete dissolution of the oil phase. Thereafter, 1 mL of potassium iodide (KI) solution was added, and the mixture was left in the dark for 10 min.

Following the incubation period, 30 mL of distilled water and 1 mL of 1% (*w*/*v*) starch solution were added. The liberated iodine was then titrated with 0.01 N sodium thiosulfate solution until the blue-black color completely disappeared. The volume of sodium thiosulfate consumed (V, mL) was recorded. The peroxide value was calculated according to the following equation: meq g O_2_/kg.

$$PV=\frac{(Vs-Vb)\times N\times 1000}{m}$$

where Vs and Vb represent the sodium thiosulfate volumes used for sample and blank titrations, respectively (mL), N is the normality of the sodium thiosulfate solution, and m is the mass of the sample (g).

Free Fatty Acid Content

The free fatty acid (FFA) content of the oil samples was determined according to the AOAC standard method at 15-day intervals throughout the 90-day storage period [23]. Briefly, 2.5–5.0 g of oil sample (m) was accurately weighed into a glass flask and dissolved in 25–50 mL of an ethanol–diethyl ether (1:1, *v*/*v*) mixture. Subsequently, 2–3 drops of phenolphthalein indicator were added, and the solution was titrated with 0.1 N sodium hydroxide (NaOH) solution until a faint pink color persisted for at least 10 s. The volume of NaOH consumed during titration (V, mL) was recorded [23]. FFA values were expressed as percentage oleic acid. The free fatty acid content was calculated using the following equation:

$$FFA\left(\%\right)=\frac{V}{m}\times 28.2$$

p-Anisidine Value

The p-anisidine value (p-AV) of the prepared oil and oleogel samples was determined at 15-day intervals throughout the 90-day storage period according to the method described by [23], based on the IUPAC method. Briefly, 0.5 g of the sample (m) was accurately weighed and diluted to a final volume of 25 mL with n-hexane. After complete dissolution, the absorbance of the solution (A1) was measured at 350 nm against n-hexane as a blank using a UV–Vis spectrophotometer (UV-160A, Shimadzu, Kyoto, Japan).

Subsequently, 5 mL of the sample solution was transferred to a test tube, and 1 mL of p-anisidine reagent was added. The mixture was allowed to react in the dark for 10 min. Thereafter, the absorbance (A2) was measured at 350 nm against a reference solution prepared by mixing 5 mL of n-hexane with 1 mL of p-anisidine reagent [23]. The p-anisidine value was calculated using the following equation:

$$p-AV=\frac{\left(1.2\times (A2-A1)\right)}{m}\times 25$$

where 25 represents the total solvent volume (mL), 1.2 is the dilution correction factor associated with the addition of the p-anisidine reagent, and m is the sample mass (g).

Fourier Transform Infrared (FTIR) Spectroscopy

The FTIR spectra of the oleogels were analyzed using an FTIR spectrophotometer (Spectrum 400, PerkinElmer Instruments, Waltham, MA, USA). Spectral data were recorded over a wavenumber range of 400–4000 cm^−1^. All analyses were performed only on freshly prepared oleogel samples.

#### 2.5.2. Cookie Analysis

Dough Color Analysis

The color parameters of the prepared cookie dough samples were determined using a precision colorimeter (FRU, precise color reader, WR-10, China). The results were expressed in terms of the CIE Lab* color space, where L* represents lightness (0 = black, 100 = white), a* represents the green (−a*) to red (+a*) axis, and b* represents the blue (−b*) to yellow (+b*) axis [17].

The total color difference (ΔE) was calculated using the following equation [11]:

$$\Delta E=\sqrt{{\left(L^{*}-{L0}^{*}\right)}^{2}+{\left(a^{*}-{a0}^{*}\right)}^{2}+{\left(b^{*}-{b0}^{*}\right)}^{2}}$$

where L*, a*, and b* represent the color parameters of the dough samples, while *L*0*, *a*0*, and *b*0* represent the corresponding color parameters of the control dough.

Dough Texture Analysis

Texture profile analysis of the cookie dough samples was performed using a Brookfield CT3 texture analyzer (Brookfield CT3, Middleborough, MA, USA) equipped with a conical probe (TA2/1000, Brookfield, Middleboro, MA, USA). All measurements were carried out at room temperature. A 1 kg load cell was used during the analyses.

The test consisted of a two-cycle penetration test to a depth of 5 mm (approximately one-third of the sample height) at a crosshead speed of 0.5 mm s^−1^. All textural parameters were automatically determined using TexturePro CT V1.9 Build 35. Each sample was analyzed in duplicate [8].

Diameter and Weight of Cookies

The physical quality characteristics of the cookie samples were evaluated by first determining their weight using an analytical balance. Subsequently, the diameter and thickness of the cookies were measured using a digital caliper.

After measuring diameter (mm) and thickness (mm), the spread ratio was calculated as the ratio of cookie diameter to thickness [24,25].

$$Spread\;ratio=\frac{Diameter\;(mm)}{Thickness\;(mm)}$$

The leavening ratio of the cookies was calculated based on the change in thickness before and after baking expressed as a percentage.

Cookie Color Analysis

The surface color parameters (L*, a*, b*) of the cookies were determined on the first day of production and during storage at 15-day intervals for up to 3 months in selected samples. Color measurements were performed using a precision colorimeter (FRU, precise color reader, WR-10, China) based on the CIE Lab* color system [13,17]. In this system, L* indicates lightness (black to white), a* represents the green (−a*) to red (+a*) axis, and b* represents the blue (−b*) to yellow (+b*) axis.

Total color difference (Δ*E*) was calculated by the following equation [11]:

$$\Delta E=\sqrt{{\left(L^{*}-{L0}^{*}\right)}^{2}+{\left(a^{*}-{a0}^{*}\right)}^{2}+{\left(b^{*}-{b0}^{*}\right)}^{2}}$$

where L*, a*, and b* are color parameters of cookies with oleogels, while *L*0*, *a*0*, and *b*0* are color parameters of control cookies.

Cookie Texture Analysis

Texture profile analysis of the cookie samples was performed using a Brookfield CT3 texture analyzer (Brookfield CT3, USA) equipped with a needle probe (TA9, Brookfield, Middleboro, MA, USA). All measurements were conducted at room temperature. The test consisted of a two-cycle penetration test to a depth of 5 mm (approximately one-third of the sample height) at a crosshead speed of 0.5 mm s^−1^. All measurements were automatically recorded using TexturePro CT V1.9 Build 35 software.

Statical Analysis

All experimental analyses were performed in triplicate. Results are presented as means ± SD (standard deviation). The differences between the means obtained in each determination were evaluated using analysis of variance (ANOVA) at a significance level of *p* ≤ 0.05, followed by Tukey’s test in IBM SPSS Statistics, Version 22 (IBM Corp., Armonk, NY, USA).

## 3. Results and Discussion

Oil-binding capacity

The oil-binding capacity (OBC) reflects the oleogel network’s ability to structure oil and is an important indicator of gel stability. Given that oleogels typically consist of up to 99% oil by weight, a high oil-binding capacity is essential to prevent oil leakage from the system and to maintain structural integrity [24].

The oil-binding capacity results of our study are given in Table 1. The oil-binding capacity results indicated no statistically significant differences among the oleogel samples (*p* > 0.05). All formulations exhibited OBC values exceeding 99%, demonstrating the oleogel network’s ability to effectively immobilize the oil phase and form a highly stable gel structure. These findings suggest that incorporating natural colorants into sunflower oil did not adversely affect the gelation behavior or oil-holding capacity of the oleogel systems.

A study [26] using oleogel as a fat substitute in cookie-making reported that oleogels structured with different oleogelators (soy wax and beeswax) exhibited OBC values above 99%, confirming the formation of stable gel networks. Among our samples, the spinach pigment-based oleogel showed the lowest OBC value, whereas the carrot pigment-based oleogel exhibited the highest value. Nevertheless, oleogels’ ability to encapsulate and immobilize oil was well preserved across all formulations, even in the presence of various functional components. This indicates that adding natural pigments to sunflower oil did not significantly influence the network-forming and oil-retention properties of the oleogels. Similar results were reported in a study investigating curcumin-loaded oleogels prepared at different beeswax concentrations, where oleogels containing 10% and 12% beeswax exhibited comparable behavior [27]. However, the authors suggested that curcumin may have influenced the gelation process, particularly at lower gelator concentrations [27].

A similar trend was reported in a study investigating oleogelation using 2% and 4% rice bran wax at different curcumin concentrations [28]. The authors observed a decrease in oil-binding capacity (OBC) with increasing curcumin concentration and suggested that this effect may be attributed to the polyphenolic nature of curcumin, which may interact with wax molecules and interfere with crystallization. In the same study, findings from carnauba wax-based oleogels indicated that incorporating curcumin did not significantly affect the oil-binding capacity. In a study investigating β-carotene-enriched oleogels [29], all prepared oleogels exhibited an oil-binding capacity (OBC) exceeding 99%. The authors reported that fish oil β-carotene incorporation did not affect the OBC of the oleogels, indicating that the oleogelators primarily determined the oil-binding capacity.

Gelation Time

Gelation time is defined as the time required to form a complete three-dimensional network within the oleogel matrix and is strongly dependent on the oleogelator concentration [30]. In the present study, no statistically significant differences were observed in gelation time among the oleogel samples (*p* < 0.05), indicating that the incorporation of natural colorant compounds in the oil phase did not affect the gelation behavior or network formation of the systems (Table 1). In addition, the gelation times obtained were within an acceptable range, suggesting that the formulations are suitable for practical application. A study on curcumin-loaded oleogels reported gelation times ranging from 23.29 to 28.03 min across different beeswax concentrations. Compared with the control, the incorporation of curcumin did not significantly affect the gelation time of the oleogels [27].

Melting point determination

Melting point is an important quality characteristic in solid fats and determines their suitability for various applications [31]. Although the control sample (CO) showed higher results than the colored oleogels, the color pigments contained in the oleogels did not affect the melting point of the oleogel (*p* < 0.05). Its high melting point makes the oleogel harder and more stable, improving the texture and overall experience of the food [32]. In the study producing oleogels and emulsifiers enriched with phenolic compounds, the melting point of the oleogels prepared with beeswax and sunflower oil was recorded at 50.42 °C. The authors reported that the presence of phenolic compounds interfered with crystallization, thus lowering the melting point. In this study, although no statistically significant difference was observed, CO showed the highest results [31]. Another study reported that the melting point of oleogels prepared with monoglycerides and phytosterols ranged from 61.75 °C to 65.65 [21]. In the study, which recorded higher melting points with increasing oleogelator concentration, it was concluded that the addition of curcumin affected the thermal behavior of the oleogel in a concentration-dependent manner [28].

Peroxide Value

An overall increase in peroxide values was observed across all samples during storage, indicating the progressive development of oxidative reactions in both oleogel and liquid oil systems.

Lipid oxidation is commonly used to evaluate the oxidative status of oils, thereby providing information on oil quality and chemical integrity. According to the Codex Alimentarius standard for edible vegetable oils, the maximum permissible peroxide value is 10 meq O_2_/kg (Codex Alimentarius Commission). In the present study, the transfer of color compounds into sunflower oil via maceration, as well as the thermal conditions applied during oleogel preparation, were considered as key factors influencing peroxide value and free fatty acid levels in both oil and oleogel systems [4].

**Figure 1:**
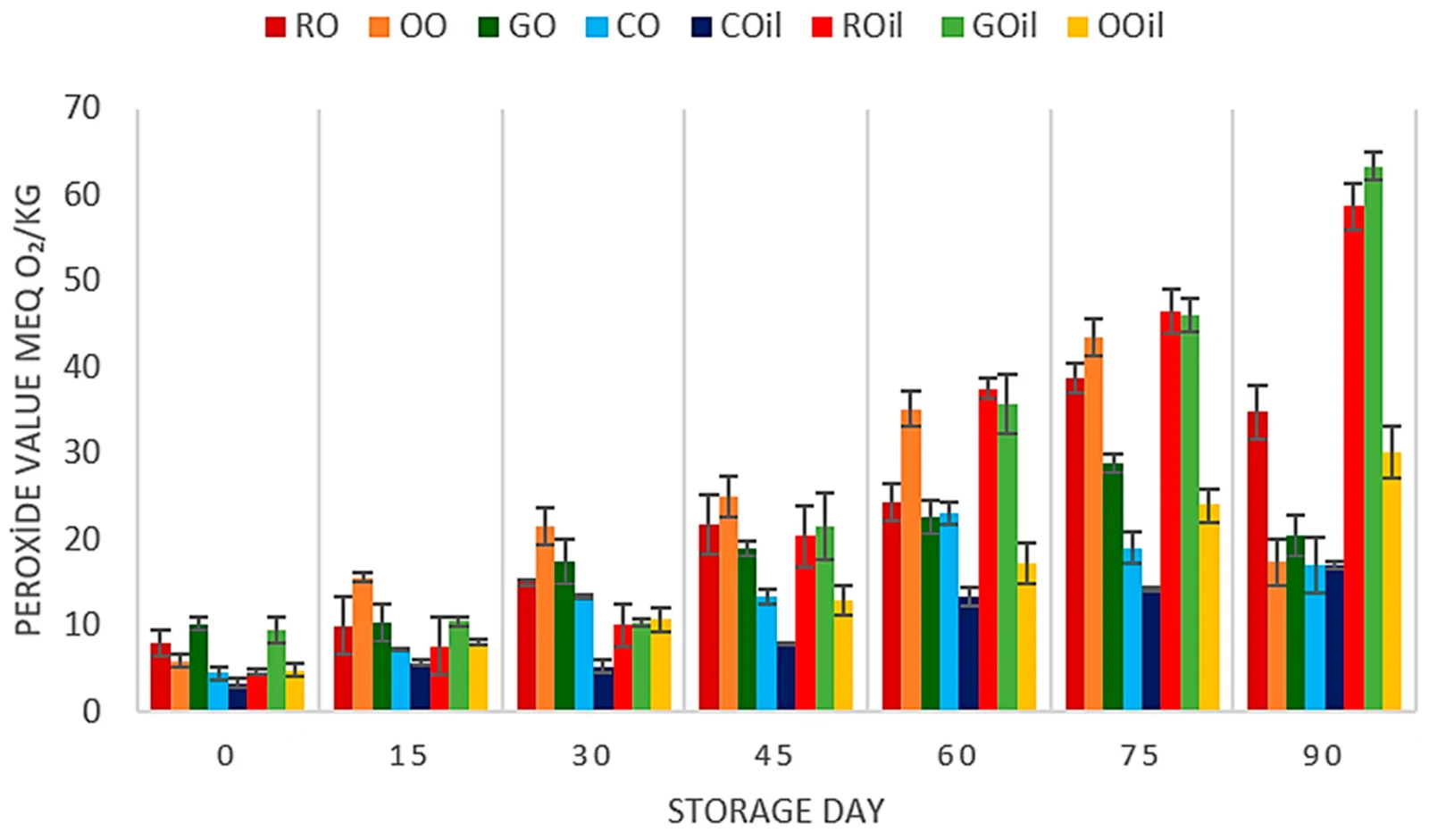
Changes in the peroxide values of oleogel and oil samples during 90 days of storage at room temperature. COil: Control oil (without colorant sunflower oil), ROil: Oil obtained by extracting tomato pigments, OOil: Oil obtained by extracting carrot pigments, GOil: Oil obtained by extracting spinach pigments, CO: Control Oleogel (without color), RO: Red Oleogel (prepared with tomato-derived color pigment-enriched oil), OO: Orange Oleogel (prepared with carrot-derived color pigment-enriched oil), GO: Green Oleogel (prepared with spinach-derived color pigment-enriched oil).

In the first 15 days, no significant increase was observed in samples other than OO (Figure 1). However, after this period, an increasing trend was observed in all samples except sunflower oil (COil). Although the increasing trend was observed in COil only after the 45th day, COil had the lowest peroxide value among the samples at the end of storage. The earlier deterioration observed in oleogel samples compared to the control COil suggests that the thermal treatment steps applied during maceration and oleogelation may have accelerated oxidative reactions. The earlier deterioration observed in the formulated samples compared with the control COil suggests that thermal processing steps during maceration and oleogelation may have contributed to the acceleration of oxidative reactions.

Although the peroxide values were initially similar, after day 45, the red and green oleogels exhibited significantly lower peroxide values than the oil from which they were prepared (*p* < 0.05). This behavior is likely associated with the structural characteristics of oleogels. The initial stage of lipid oxidation involves the formation of primary oxidation products such as hydroperoxides through the reaction of fatty acids with oxygen [4]. One of the key advantages of oleogels is their ability to enhance the oxidative stability of oils through structural organization. The oleogel network may restrict oxygen diffusion within the lipid matrix, thereby reducing the rate of lipid oxidation [33]. As reported by Habibzadeh et al. [34], oleogelation can enhance oil retention by restricting oil mobility and limiting oxygen diffusion within the matrix, thereby slowing oxidative processes and improving storage stability. In addition, the presence of bioactive compounds in the oil phase may further influence oxidative pathways by acting as promoters of oxidation under specific conditions.

OO showed a higher peroxide value than OOil during storage. Accordingly, it can be concluded that beta-carotene has no effect in preventing or delaying oxidation. Studies show that β-carotene alone does not provide significant oxidative protection in oleogel [35]. Furthermore, carotenoids have been reported to exhibit pro-oxidant activity under certain conditions [36,37], while chlorophyll derivatives such as pheophytins may also act as photosensitizers, promoting oxidation in the presence of light [9,38]. The occasional lower peroxide values observed in COil suggest that the addition of plant-derived pigments does not universally enhance oxidative stability. Instead, their effect may vary with storage conditions and system composition, and may even contribute to pro-oxidant behavior during long-term storage.

Similarly, a study [33] investigating the addition of tomato-derived components to sunflower oil [39] reported an increase in peroxide values, ranging from 4.30 to 4.58, consistent with the trends observed in the present work. In another study comparing beeswax-based oleogels and sunflower oil systems [33], sunflower oil exhibited the lowest primary oxidation values, in agreement with the present findings. In contrast, peroxide values increased throughout storage across all samples, with the lowest values consistently observed in the control oil.

Free Fatty Acid

**Figure 2:**
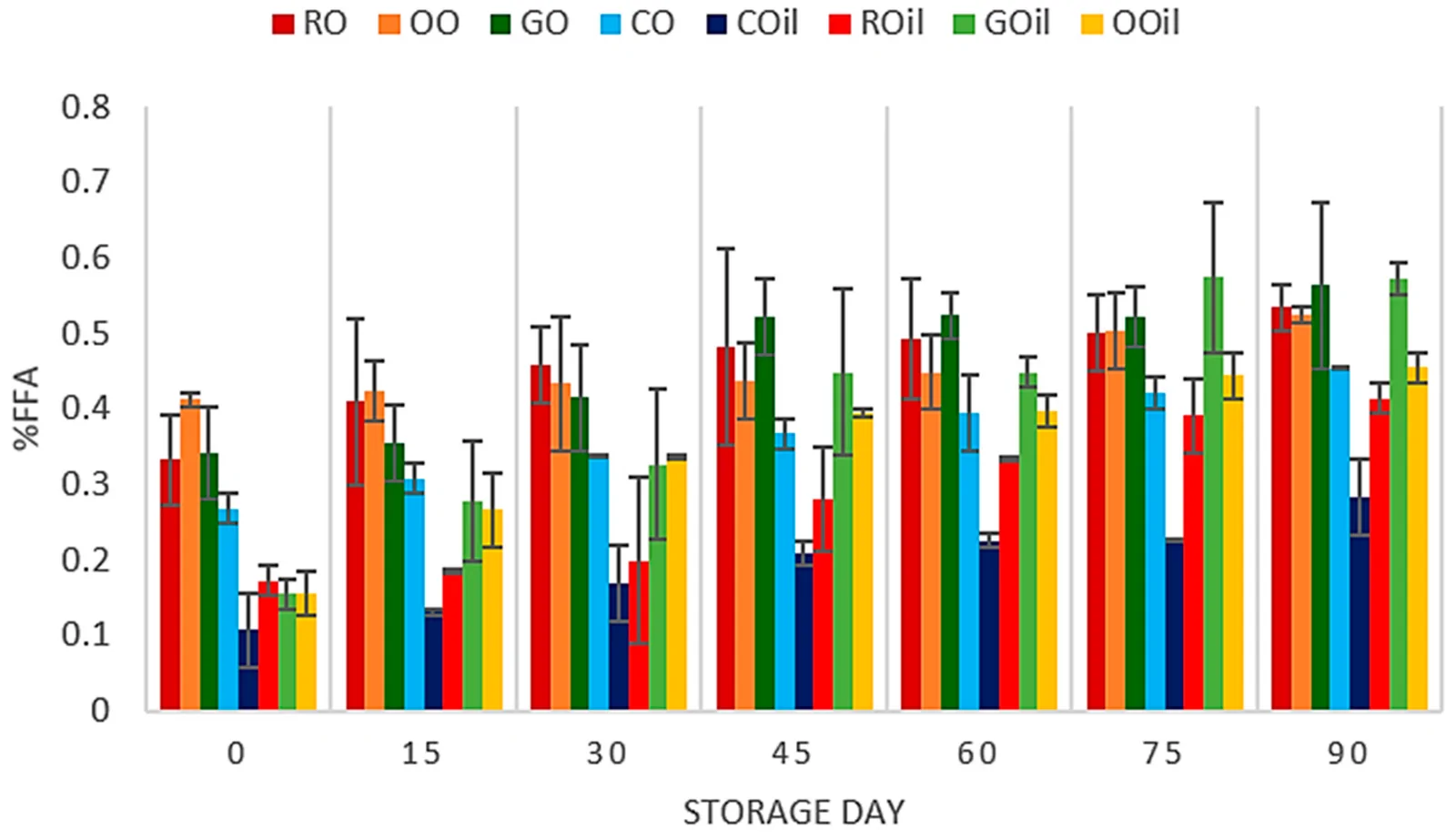
Changes in the free fatty acid content of oleogel and oil samples during 90 days of storage at room temperature. COil: Control oil (without colorant sunflower oil), ROil: Oil obtained by extracting tomato pigments, OOil: Oil obtained by extracting carrot pigments, GOil: Oil obtained by extracting spinach pigments, CO: Control Oleogel (without color), RO: Red Oleogel (prepared with tomato-derived color pigment-enriched oil), OO: Orange Oleogel (prepared with carrot-derived color pigment-enriched oil), GO: Green Oleogel (prepared with spinach-derived color pigment-enriched oil).

According to the results of free fatty acid (FFA) analysis performed every 15 days for a period of 90 days, while the initial values of colored oleogels and oils were very close to each other, GO surpassed other oleogels from day 45 onwards, showing the highest FFA value in subsequent storage days (Figure 2). Among colored oleogels, OO had the highest initial FFA value but the lowest FFA on the last day of storage. Similarly, in oils, GOil stood out from other oils, recording a higher FFA result on day 45. The lowest FFA was recorded in COil throughout storage. FFA content increased in all products throughout storage.

A study indicated that the high FFA content in oleogels may be related to the breakdown of free fatty acids and/or wax esters contained in natural waxes [40]. This could be why the oleogels in our study exhibited a higher FFA value than the oils from which they were prepared. The fact that COil exhibited the lowest FFA value throughout storage also supports this view. Furthermore, in another study, the authors reported that the FFA content increased in oils exposed to heat, and, in parallel with our study, the increase in FFA in oleogels at the end of storage was higher than in oil [41].

According to regulatory limits in several European countries, acceptable FFA values for edible oils typically range between 0.9% and 2.5% [42]. In this context, all samples remained within the permissible range even after 90 days of storage, indicating acceptable chemical quality throughout the storage period.

p-Anisidine Value

**Figure 3:**
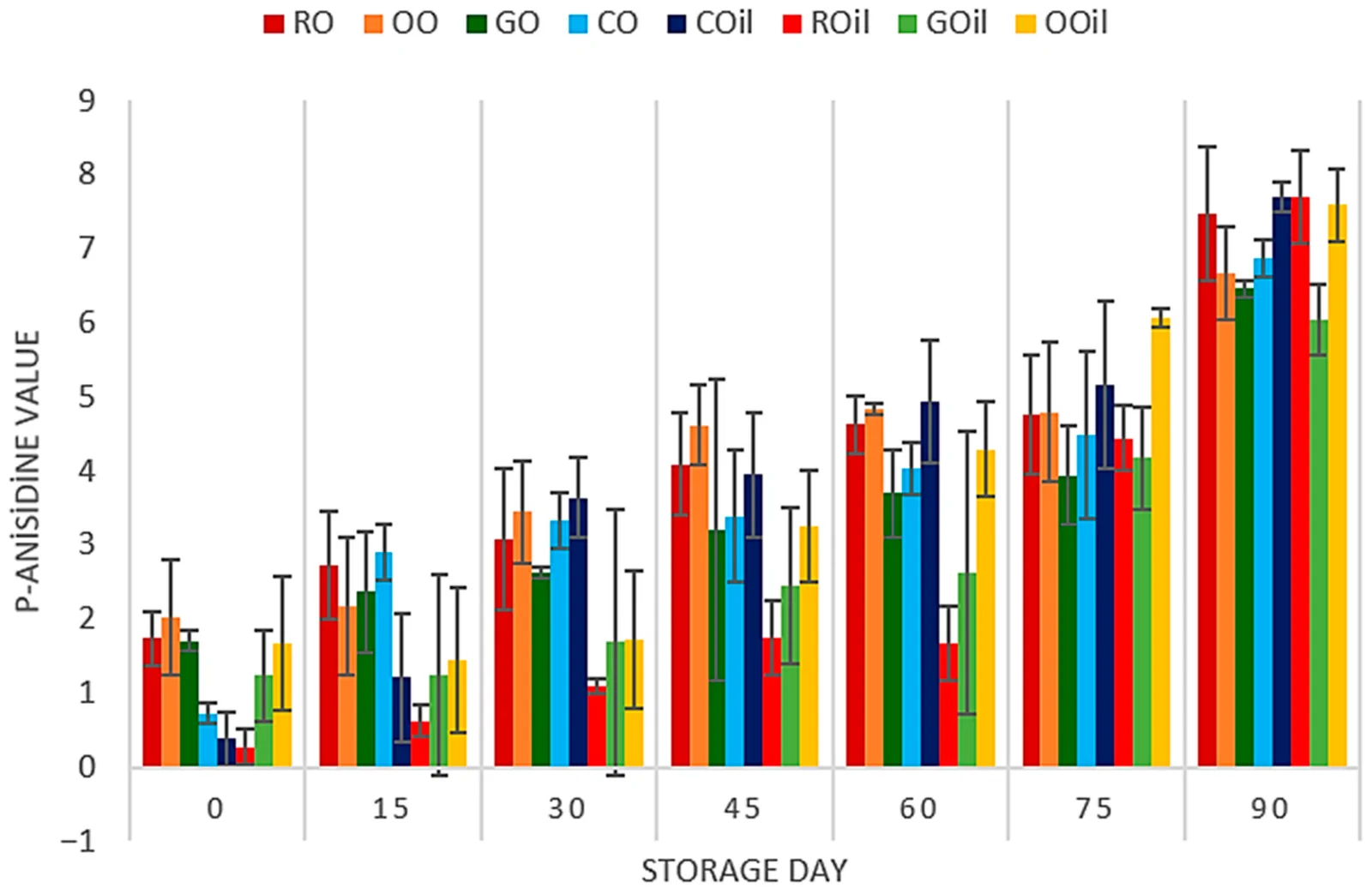
Changes in the p-anisidine values of oleogel and oil samples during 90 days of storage at room temperature. COil: Control oil (without colorant sunflower oil), ROil: Oil obtained by extracting tomato pigments, OOil: Oil obtained by extracting carrot pigments, GOil: Oil obtained by extracting spinach pigments, CO: Control Oleogel (without color), RO: Red Oleogel (prepared with tomato-derived color pigment-enriched oil), OO: Orange Oleogel (prepared with carrot-derived color pigment-enriched oil), GO: Green Oleogel (prepared with spinach-derived color pigment-enriched oil).

The evaluation of secondary oxidation products, particularly aldehydic compounds, is commonly assessed using the p-anisidine value (p-AV) [33]. COil showed a significantly higher pAV value than the colored oils on day 30, and the values approached each other again in the last days of storage (Figure 3). Accordingly, it can be concluded that the color pigments added to the oils delayed secondary oxidation during storage. This result is also supported by studies showing that color pigments added to the oil slow down secondary oxidation [43,44,45]. All oleogels exhibited similar behavior during storage. In contrast, CO, which gave the lowest result among the oleogels in the initial day analysis, showed a similar increasing trend with the other oleogels in the following days of storage. Accordingly, the oleogelation mechanism, rather than the added color pigments, may affect the pAV results of the oleogels. In all colored samples, oleogels showed higher pAV values than the oils from which they were prepared. This suggests that it may be associated with processing conditions applied during oleogel preparation. Similar effects of heat-induced secondary oxidation have been reported in previous studies [46]. Higher p-AV in oleogels have been attributed to the heat treatment applied during oleogel preparation. Applied temperatures may trigger the decomposition of hydroperoxides into secondary oxidation products, thereby increasing PAV values.

When evaluated on a product basis, p-anisidine values showed considerable variation among samples; however, this behavior is consistent with the typical response of secondary oxidation indices during storage [33]. A noticeable increase was observed across all samples on day 90, suggesting an acceleration in the formation of secondary oxidation products toward the end of the storage period. According to established quality criteria, p-anisidine values below 10 are considered acceptable for edible oils [47]. In the present study, all samples remained below this threshold throughout the 90-day storage period, indicating that their oxidative quality remained within acceptable limits despite the observed increase over time.

Color Properties

**Figure 4:**
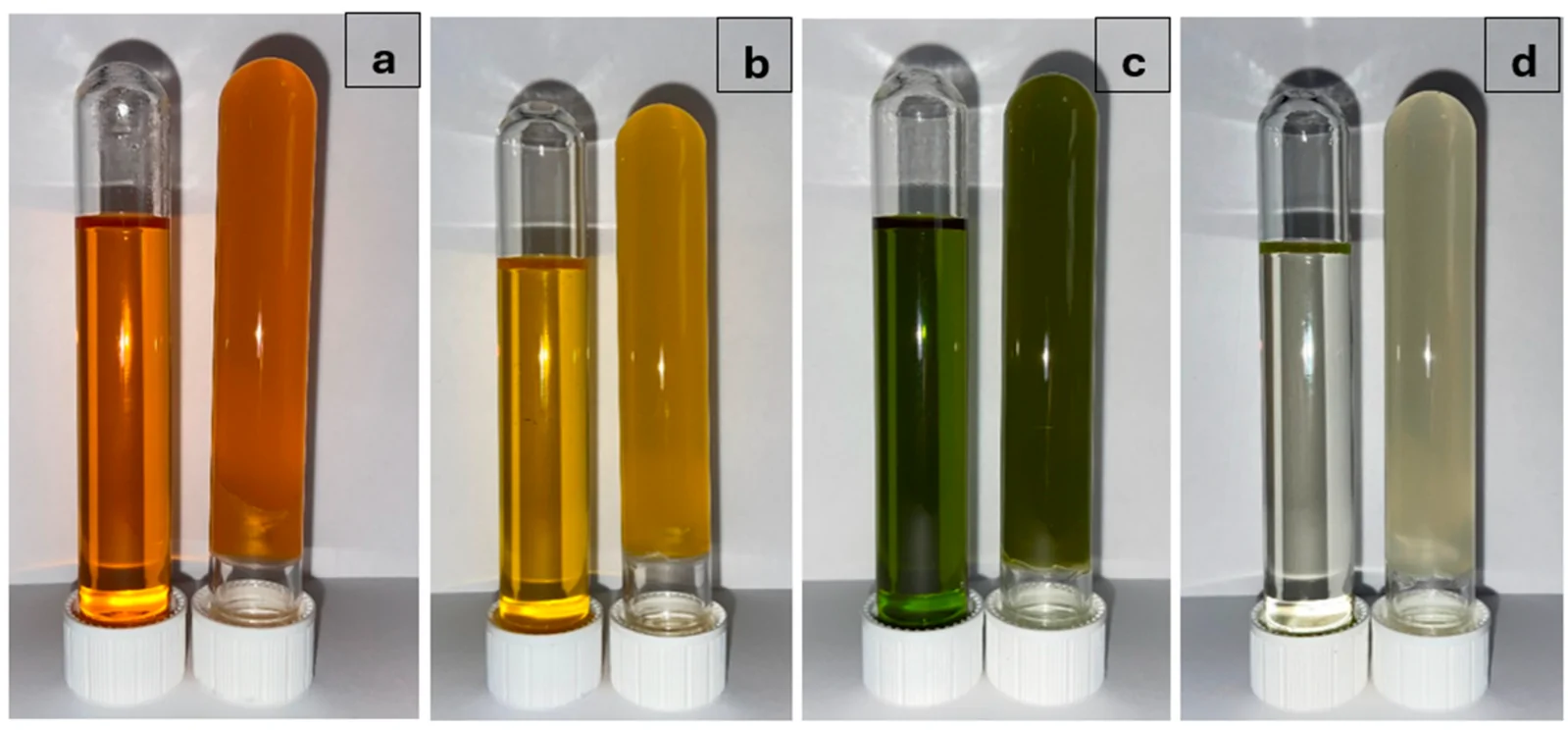
Appearance of the oil and oleogel samples: (**a**) Oil enriched with tomato-derived color pigments and the red oleogel prepared using this enriched oil; (**b**) Carrot-derived color pigment-enriched oil and the orange oleogel prepared using this enriched oil; (**c**) Spinach-derived color pigment-enriched oil and the green oleogel prepared using this enriched oil; (**d**) Sunflower oil and control oleogel prepared with only sunflower oil without color pigment.

Color is an important quality parameter in determining the overall acceptability and consumer perception of final food products [2]. Despite ongoing arguments about the harmful effects of artificial colorings, they are widely used in the food industry, including baked foods. On the other hand, naturally grown fruits, vegetables, and plants contain a wide variety of fat-soluble color pigments. In our study, tomatoes, carrots, and spinach, which are rich in red, orange, and green color pigments, respectively, were selected as the aforementioned color sources. Instead of using organic solvents to extract the targeted color pigments from these sources, vegetable oil, which readily dissolves them, was used (Figure 4). Due to the health risks associated with organic solvents, vegetable oils are used as green solvents for extracting fat-soluble substances in many areas. In our study, colored oleogels produced from the colored oils were used as an alternative to solid margarine used in cookie production. In cookies produced with oleogels colored with color pigments obtained from tomatoes, carrots, and spinach, red, orange, and green colors were observed, respectively.

The colored oil samples differed significantly from each other in terms of ΔE* (*p* < 0.05) (Table 2). The pronounced ΔE* values observed in the present study can be attributed to the substantial changes in the L*, a*, and b* coordinates following the incorporation of the colorants. Previous studies have demonstrated that the color of edible oils is strongly influenced by their pigment composition, which can markedly affect the L*, a*, and b* coordinates [48]. In particular, differences in pigment content have been associated with significant variations in the redness and yellowness of vegetable oils. Therefore, the high ΔE* values obtained for the colored oils in the present study indicate a substantial alteration in color compared with the control oil, most likely due to the strong contribution of the added colorants to the overall color characteristics of the oil phase.

The color difference (ΔE*) values of the oleogel samples relative to the control oleogel were 35.23, 25.81, and 15.68, respectively, indicating pronounced color differences among the samples (Table 3). Since ΔE* values above 3 are generally considered perceptible to the human eye, all oleogel samples exhibited visually distinguishable color differences compared with the control oleogel [49]. Similar findings have been reported in previous studies, where substantial color differences were observed among oleogels prepared with different structuring agents. For instance, ΔE* values ranging from 42.20 to 53.04 were reported for rapeseed oil-based oleogels, with the authors attributing the differences to the type of structuring agent and its effect on the color characteristics of the oleogel system [49]. In another study, ΔE* values of 12.74–28.08 were reported for different oleogel formulations relative to a reference sample, further demonstrating that oleogel composition can substantially influence color characteristics [50]. Therefore, the relatively high ΔE* values observed in the present study may be associated with differences in oleogel composition and the presence of the added colorants, which altered the L*, a*, and b* coordinates relative to the control oleogel.

The relatively high color difference observed between the oil and its corresponding oleogel (Table 4) may be associated with changes in the optical properties of the system following oleogelation. The formation of a three-dimensional network and the presence of dispersed crystalline structures can alter light scattering and consequently affect the L, a*, and b* values. Similar substantial color differences have been reported for oleogel systems prepared using different structuring agents [49].

The color of oleogels is primarily governed by the combined contribution of the raw materials used in their formulation. In the present study, no statistically significant differences were observed among samples during storage within each formulation (*p* > 0.05). All oleogel samples exhibited lower L* values compared to their corresponding liquid oils, which can be attributed to the presence of the oleogelator and the structural changes induced by the oleogelation process.

A similar trend has been reported in the literature. In a study investigating oleogels prepared with different waxes (carnauba wax, beeswax, and candelilla wax), the highest L* values were observed in candelilla wax-based oleogels [51]. Likewise, another study reported that incorporating 3% beeswax into butia seed oil led to a noticeable reduction in L* values compared with the original oil, confirming the effect of oleogelation on color parameters [4].

**Figure 5:**
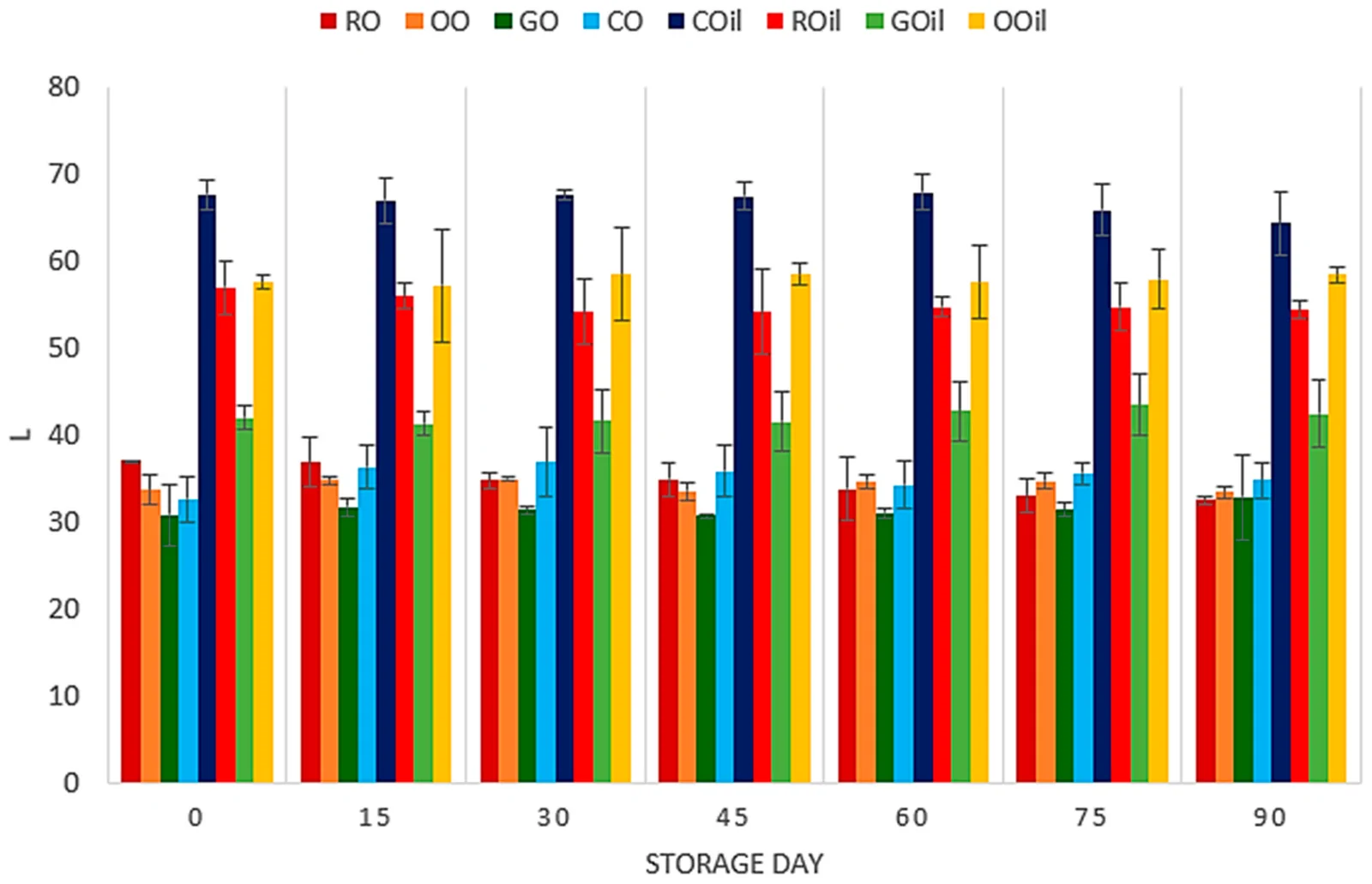
Changes in the L values of oleogel and oil samples during 90 days of storage at room temperature. COil: Control oil (without colorant sunflower oil), ROil: Oil obtained by extracting tomato pigments, OOil: Oil obtained by extracting carrot pigments, GOil: Oil obtained by extracting spinach pigments, CO: Control Oleogel (without color), RO: Red Oleogel (prepared with tomato-derived color pigment-enriched oil), OO: Orange Oleogel (prepared with carrot-derived color pigment-enriched oil), GO: Green Oleogel (prepared with spinach-derived color pigment-enriched oil).

In the present study, no statistically significant differences in L* values were observed between colored oleogels and the control oleogel (CO) throughout the entire storage period (*p* > 0.05). Among all samples, COil consistently exhibited the highest L* values at all storage time points (Figure 5). These findings suggest that neither the incorporation of natural pigments nor storage duration significantly affected the lightness of the oleogel systems.

Consistent with these results, a study in which lycopene was incorporated into coconut oil-based oleogels reported L* values of 36.33 for the control oleogel and 38.24 for the lycopene-enriched oleogel (RO) [52]. In another study, oleogels prepared by adding 3% candelilla wax to flaxseed oil exhibited color parameters of L* = 42.53, a* = −1.64, and b* = 16.19 [2], while chia oil oleogels containing 8% beeswax showed L* = 50.42, a* = −2.97, and b* = 16.72 [27]. The color values obtained in the present study for the control oleogel (CO) are in good agreement with these findings. Minor variations among studies can be attributed to differences in the type of liquid oil used and variations in oleogel formulation and processing conditions.

The differences in a* values among the samples are primarily associated with the type of natural pigments extracted from the plant sources. Tomato-derived pigments, mainly lycopene, contributed to the higher positive a* values, whereas spinach-derived chlorophyll promoted negative a* values, indicating a shift toward greener tones.

**Figure 6:**
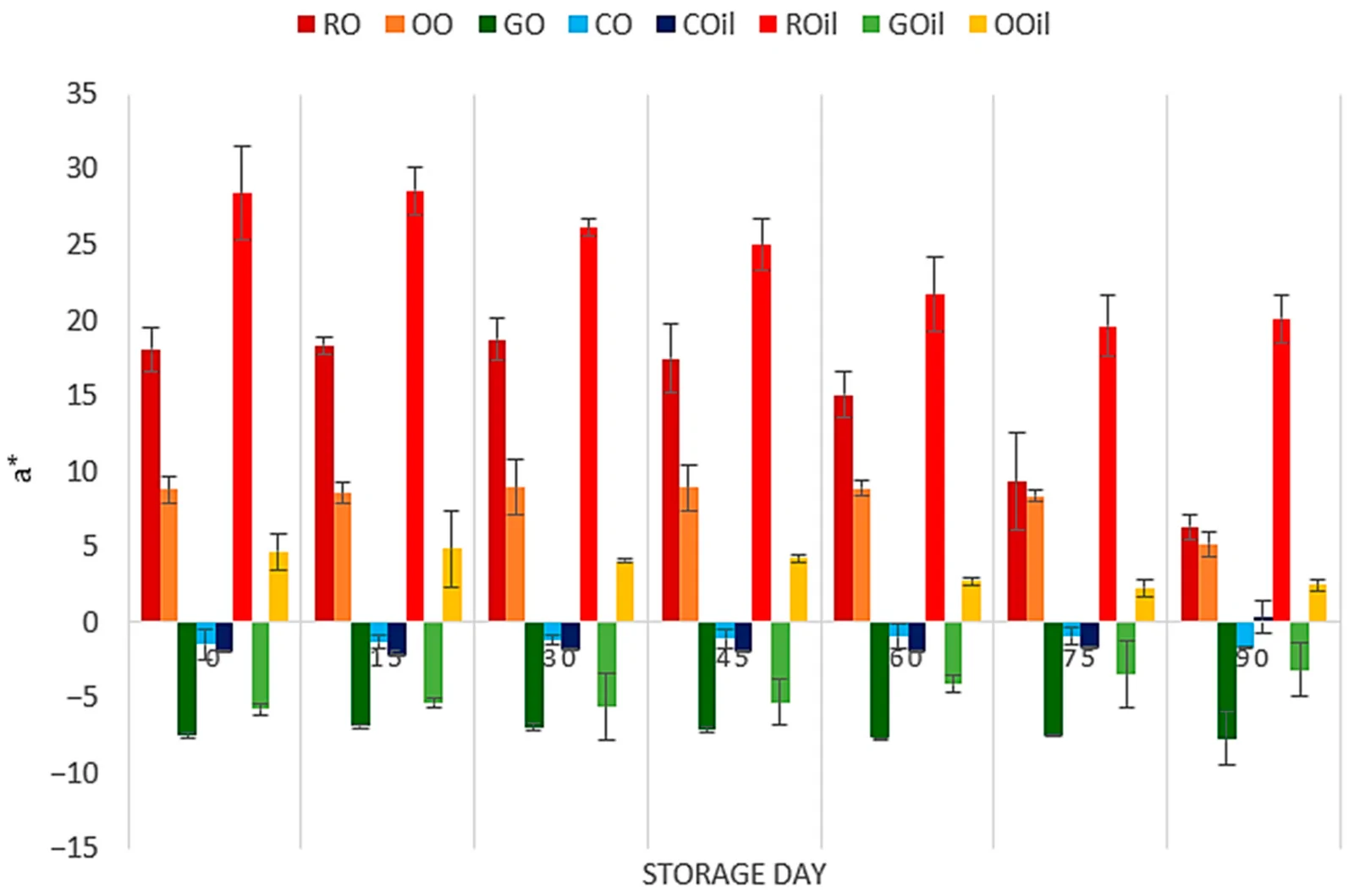
Changes in the a* values of oleogel and oil samples over 90 days of storage at room temperature. COil: Control oil (without colorant sunflower oil), ROil: Oil obtained by extracting tomato pigments, OOil: Oil obtained by extracting carrot pigments, GOil: Oil obtained by extracting spinach pigments, CO: Control Oleogel (without color), RO: Red Oleogel (prepared with tomato-derived color pigment-enriched oil), OO: Orange Oleogel (prepared with carrot-derived color pigment-enriched oil), GO: Green Oleogel (prepared with spinach-derived color pigment-enriched oil).

In color analysis, the a* value was considered a critical parameter for evaluating the red and green color characteristics of the samples examined in this study. Based on a* values, the highest redness intensity was observed in ROil, followed by RO; OO and OOil exhibited moderate a* values. These samples exhibited a more pronounced reddish appearance, with significantly higher a* values compared to CO and COil (Figure 6). Therefore, the results showed that the highest redness was obtained in ROil, followed by RO. Although RO exhibited a high redness value, the slightly higher a* value of ROil suggests that the oleogel network formation may partially affect the visual expression of tomato-derived pigments by altering light scattering within the matrix.

As expected, GO and GOil exhibited green color characteristics, with GOil reaching negative a* values, confirming the dominance of green tones in this sample (Figure 6). The observed color differences among the oleogels can be attributed to the oleogel former’s effect on the system’s optical properties and structural organization. Furthermore, the comparison between GOil and COil revealed that GOil’s lower a* value was associated with a more intense green color, indicating that spinach-derived pigments contribute to the green appearance of the oil-based system.

The differences in color values of oils and their corresponding oleogels may be related to the formation of a three-dimensional oleogel network. The oleogelator-induced structural organization can modify pigment dispersion and light scattering behavior, resulting in different color perceptions compared with the original oils. Table 4 presents the color differences between oleogels and the oils that make up the oleogel. As can be seen here, oleogel formation caused a significant change in color difference.

From a colorimetric perspective, the GOil exhibited negative a* values throughout the storage period, which was consistent with its characteristic green color. No statistically significant changes were observed in this parameter over time, indicating good color stability. The highest a* values were recorded in the ROil samples, followed by the RO.

In a previous study, lycopene-enriched oleogels prepared with coconut oil showed an increase in a* values from 0.01 in the control sample to 1.45 in the lycopene-fortified formulation [52]. These findings are in agreement with the present study, particularly for the CO and RO systems. The relatively high a* values observed in the present work may be attributed to the presence of lycopene as well as the structuring effect of the oleogel matrix. The same study also reported that decreasing lycopene concentration led to a reduction in a* values [52], which is consistent with the significant decrease in a* values observed in the present study after day 60, suggesting a reduction in lycopene content over storage.

A marked decline in a* values in both ROil and RO after 60 days indicates the degradation of lycopene during storage. Notably, even at the final storage, the a* value of ROil remained higher than that of RO, which may be attributed to processing conditions and the influence of the oleogel network on pigment stability. The observed decrease in redness after day 60 suggests that the shelf life of lycopene-associated color intensity was approximately 45 days, after which pigment degradation became more pronounced.

Considering the relationship between oxidation and color changes, the increase in peroxide values after 30 days supports the hypothesis that oxidative degradation may have contributed to the breakdown of lycopene in the system. When a* values are evaluated together with peroxide values, it can be suggested that lycopene-containing systems may have exhibited pro-oxidant behavior during extended storage.

Consistent with the present findings, a study involving oleogels prepared with 5% beeswax and canola oil reported negative a* values (−3.78) [8]. In the same study, incorporating β-carotene at different concentrations resulted in a* values ranging from 9.72 to 20.35, which aligns well with the behavior observed in the carrot-based oleogel in the present work.

One of the notable findings of this study was the remarkable stability of the GO, which showed no significant change in a* values over the 90-day storage period. Chlorophyll pigments are highly susceptible to degradation under acidic conditions, heat, light, and oxygen. Under thermal conditions, magnesium in the chlorophyll structure is replaced by hydrogen ions, leading to the formation of pheophytins and a consequent loss of green color [53]. Additionally, chlorophyllase activity can catalyze the removal of the phytol chain, forming chlorophyllide and further contributing to color loss. The optimum temperature range for chlorophyllase activity is reported to be between 60 °C and 82.2 °C, with denaturation occurring at 100 °C [53]. Although both maceration and oleogelation processes involved temperatures within this range, no significant color degradation was observed, and the green color was retained throughout 90 days of storage at room temperature.

These results suggest that the oleogel matrix effectively protected chlorophyll pigments against thermal, oxidative, and enzymatic degradation, demonstrating the potential of oleogel systems as protective carriers for sensitive bioactive color compounds.

**Figure 7:**
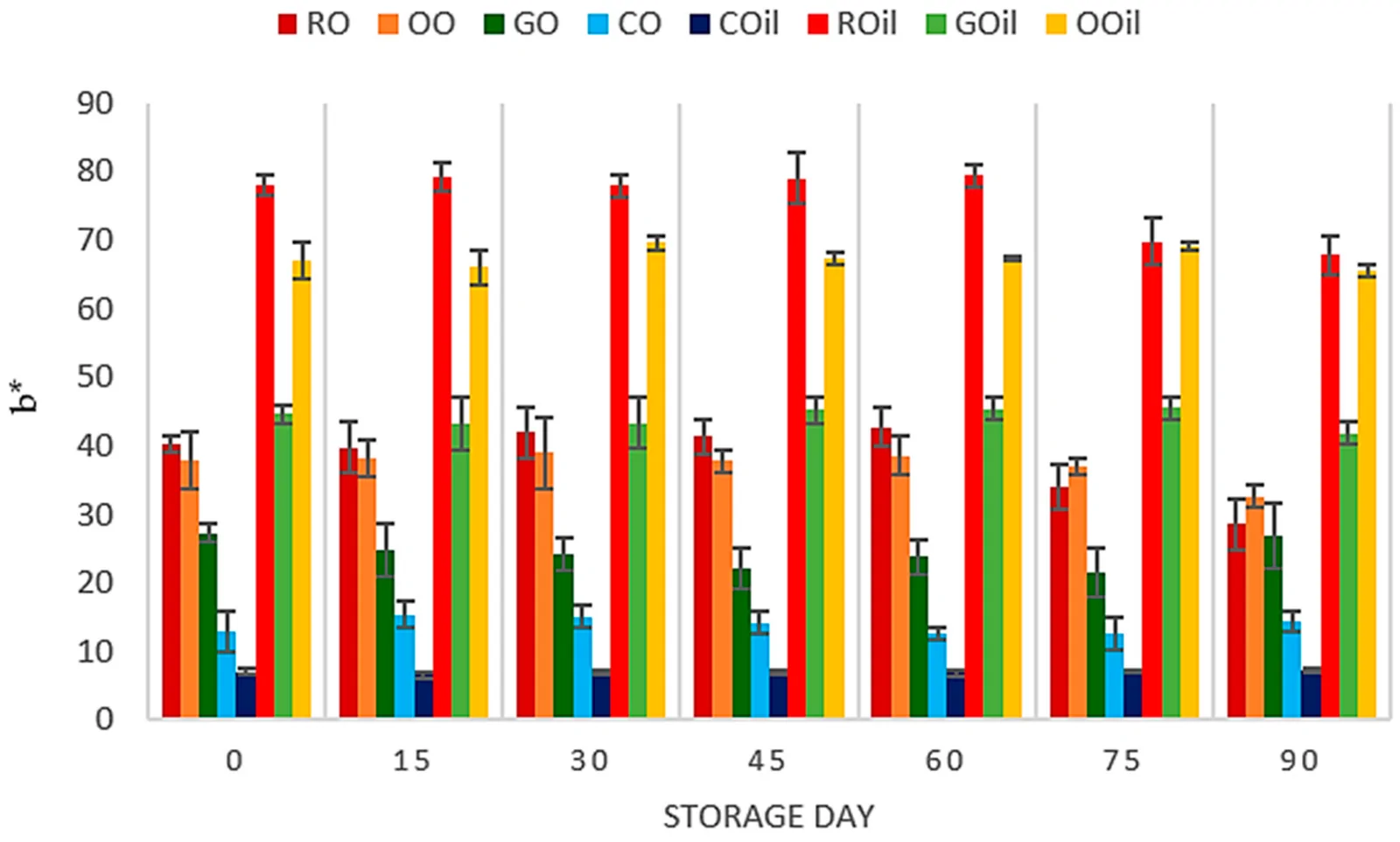
Changes in the b* values of oleogel and oil samples during 90 days of storage at room temperature. COil: Control oil (without colorant sunflower oil), ROil: Oil obtained by extracting tomato pigments, OOil: Oil obtained by extracting carrot pigments, GOil: Oil obtained by extracting spinach pigments, CO: Control Oleogel (without color), RO: Red Oleogel (prepared with tomato-derived color pigment-enriched oil), OO: Orange Oleogel (prepared with carrot-derived color pigment-enriched oil), GO: Green Oleogel (prepared with spinach-derived color pigment-enriched oil).

The highest b* values were observed in ROil and OOil, whereas the lowest values were recorded in the CO and COil samples (Figure 7). Overall, no statistically significant changes in b* values were detected during storage for most samples; however, a significant decrease was observed in the RO at days 75 and 90. A similar change has also been observed in ROil.

In a previous study investigating oleogels enriched with different levels of β-carotene, b* values ranged from 10.38 in the control sample to 3.91–28.75 in carotene-enriched formulations [8]. These results are in agreement with the behavior observed in the OO in the present study, confirming the strong contribution of carotenoid pigments to the yellow color intensity of oleogel systems.

Overall, the obtained color parameters confirmed that pigment-enriched oils successfully transferred their characteristic colors to oleogel systems. The ability to produce red, orange, and green oleogels demonstrates the potential of plant-derived pigments as natural color sources for designing functionally and visually appealing lipid-based food systems.

Hardness

**Figure 8:**
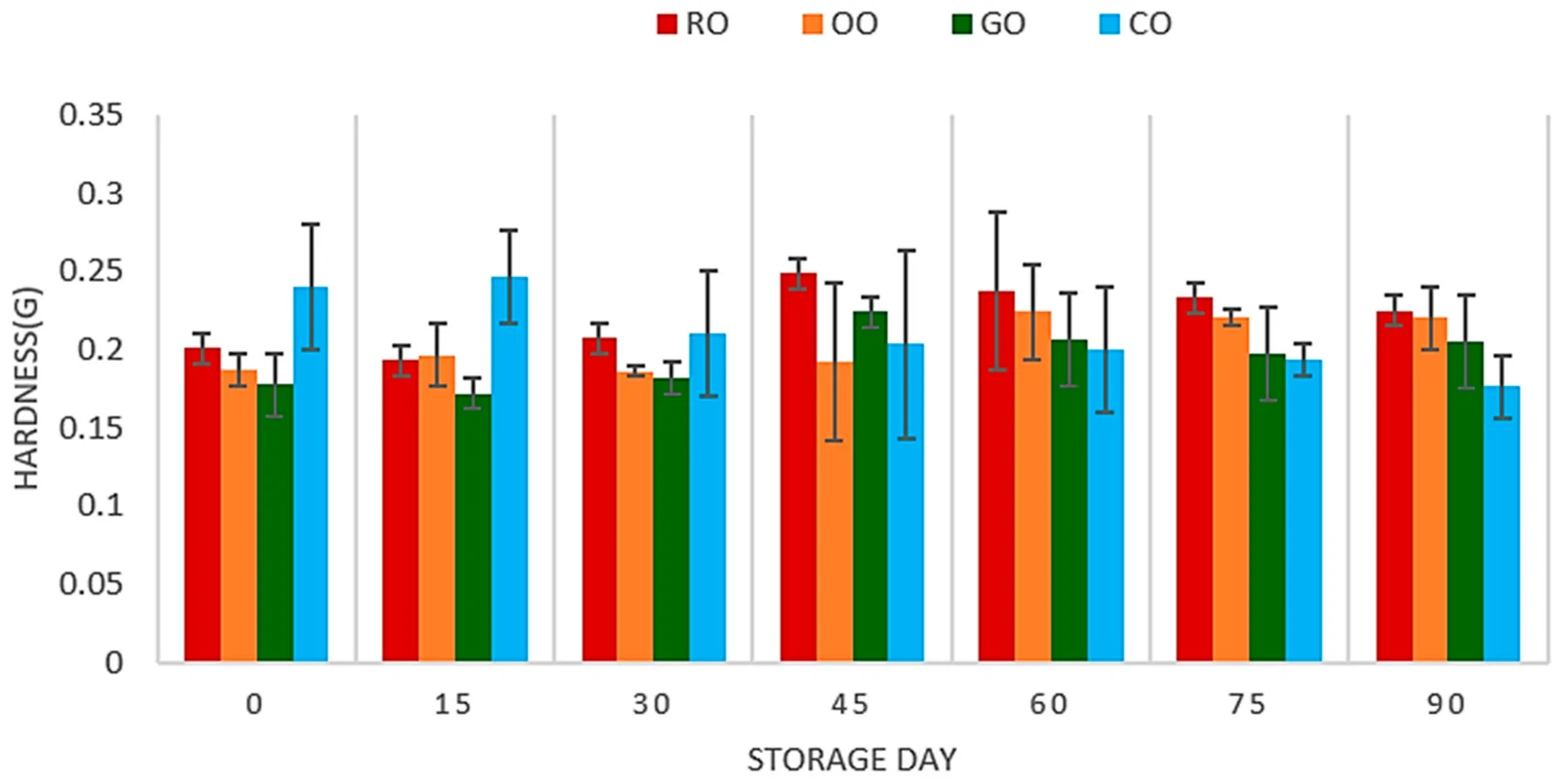
Changes in the hardness of oleogel samples during 90 days of storage at room temperature. CO: Control Oleogel (Without color), RO: Red Oleogel (prepared with tomato-derived color pigment-enriched oil), OO: Orange Oleogel (prepared with carrot-derived color pigment-enriched oil), GO: Green Oleogel (prepared with spinach-derived color pigment-enriched oil).

When comparing the hardness of oleogels during storage, no statistically significant difference was observed among samples (*p* > 0.05). The control oleogel showed a decrease in hardness over the later days of storage, while the colored oleogels showed an increase in hardness (Figure 8). In a study that added curcumin to oleogels, it was reported that curcumin slightly increased the stiffness of the oleogels and had a slight effect on their mechanical strength [54]. It has been reported that increasing the amount of beta-carotene will reduce the hardness of the oleogel [55]. In the study reported by [22], higher hardness values were associated with oleogel preparation techniques involving high-speed homogenization, whereas in the present study, which employed a conventional preparation method, comparable hardness values (0.143–0.171 g) were obtained. This finding indicates the positive structuring effect of candelilla wax on texture development.

FTIR

**Figure 9:**
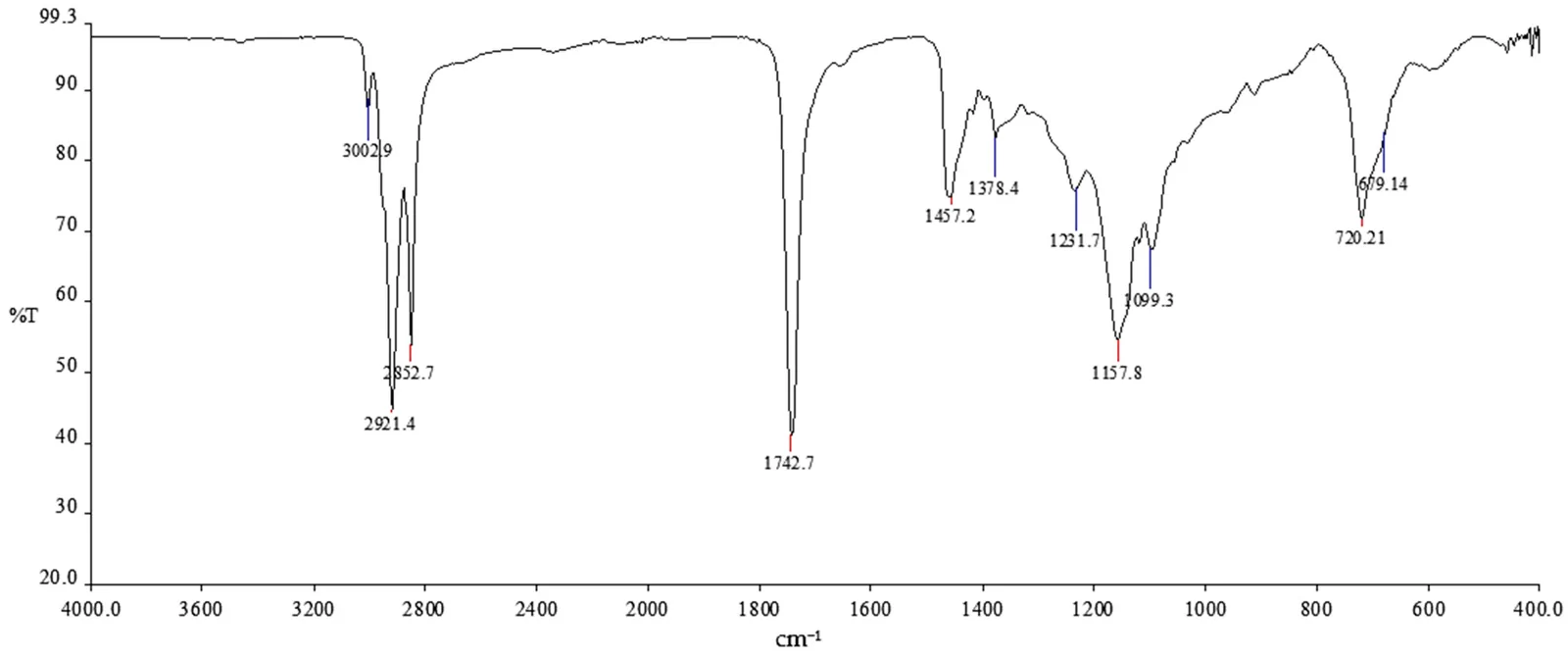
FTIR spectra of red oleogel (RO).

**Figure 10:**
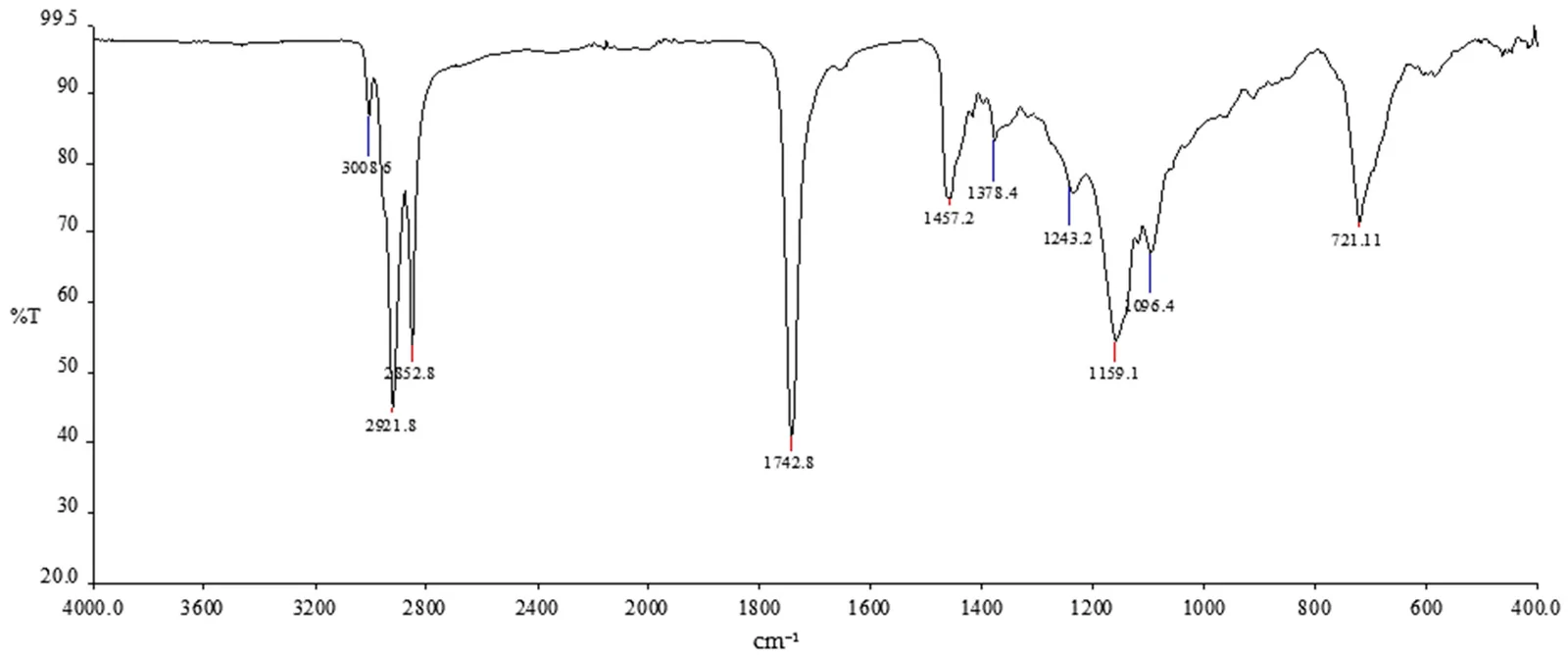
FTIR spectra of green oleogel (GO).

**Figure 11:**
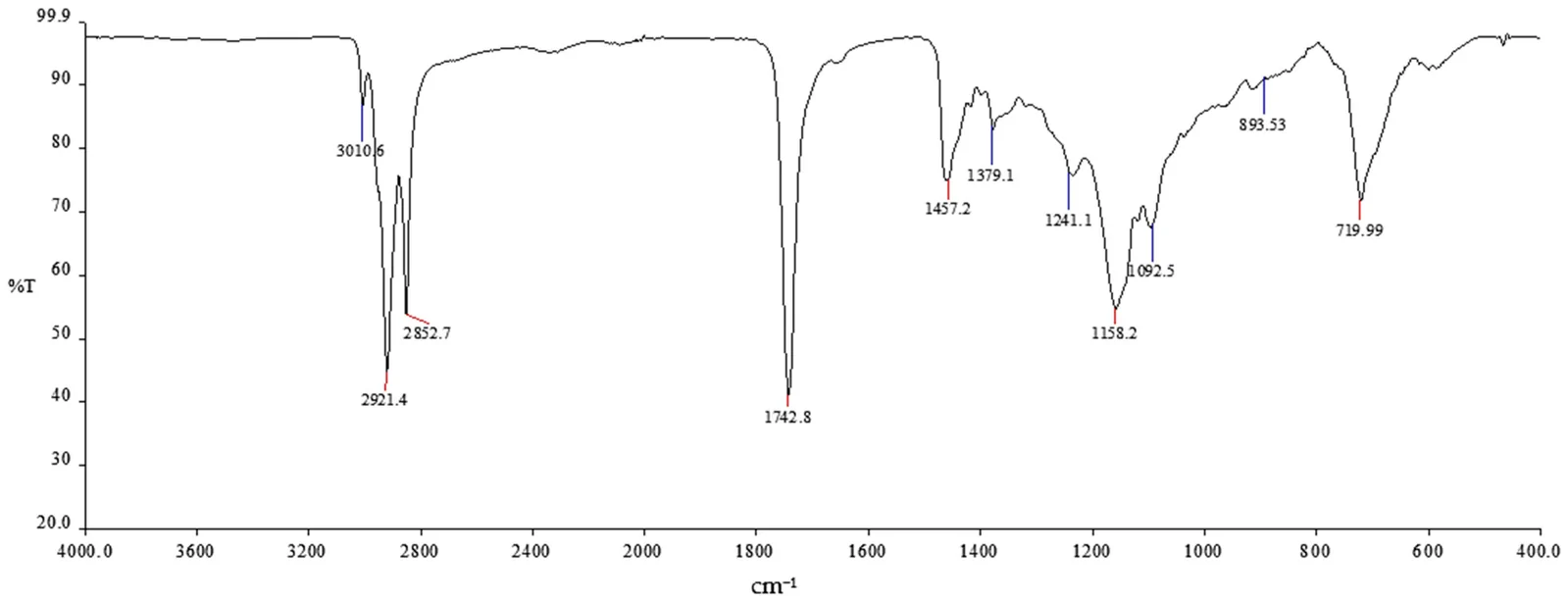
FTIR spectra of orange oleogel (OO).

**Figure 12:**
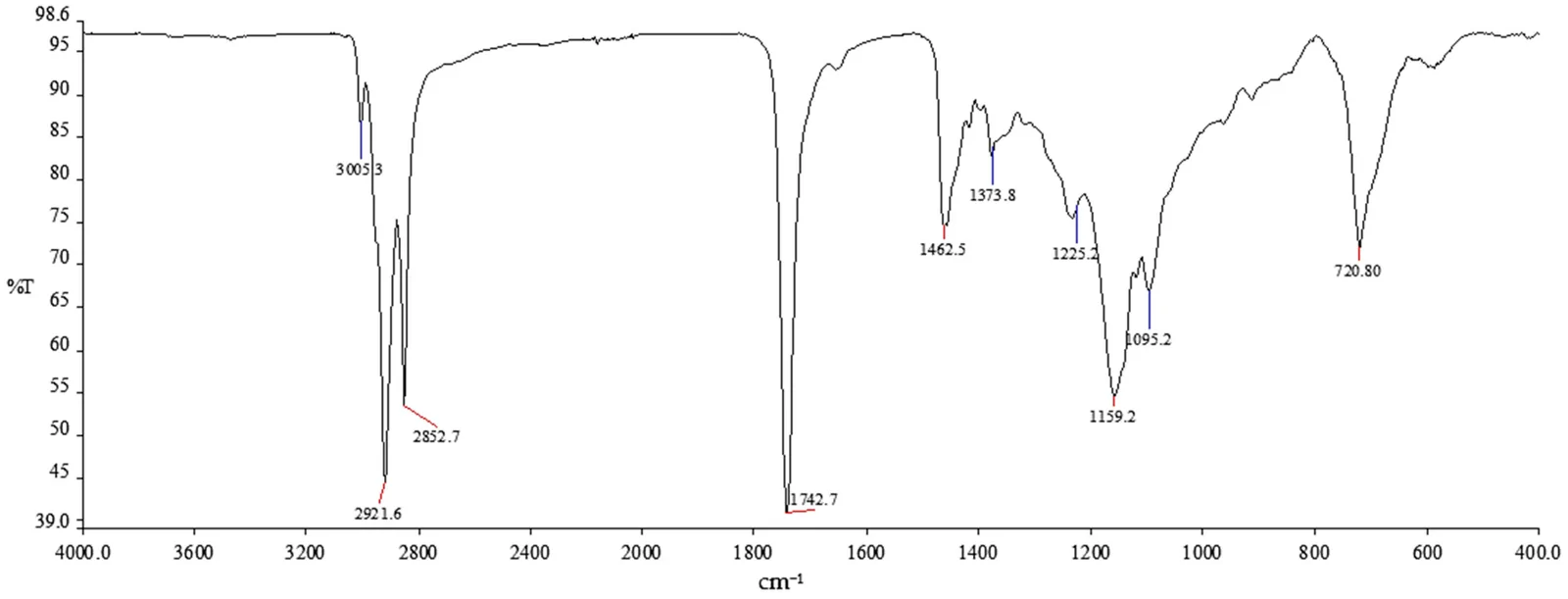
FTIR spectra of control oleogel (CO).

FTIR results not only confirm the chemical identity of the oleogel systems but also provide insight into the molecular interactions governing their structural organization. The absence of new peaks or significant shifts in characteristic bands across all formulations indicates that the oleogelation process and the incorporation of natural colorant compounds did not lead to the formation of new covalent bonds or chemical modifications within the lipid matrix (Figure 9, Figure 10, Figure 11 and Figure 12).

The strong absorption band observed at approximately 1742 cm^−1^ corresponds to the ester carbonyl (C=O) stretching vibration of triglycerides, confirming the dominance of lipid-based ester structures in the system [22]. The presence of aliphatic C–H stretching bands at 2921–2922 cm^−1^ and 2852–2853 cm^−1^ further supports the integrity of long-chain hydrocarbon structures, indicating that the triglyceride backbone remained chemically stable throughout the oleogelation process [8,56]. In addition, the C–O stretching vibrations detected between 1090 and 1240 cm^−1^ reinforce the presence of ester functionalities and confirm the preservation of the native oil chemical framework [22].

From a structural perspective, these findings suggest that oleogel formation is governed predominantly by physical interactions rather than chemical reactions. In particular, wax-based oleogelators are expected to form a three-dimensional network through weak intermolecular forces such as van der Waals interactions and hydrophobic associations, thereby immobilizing the liquid oil phase without altering its fundamental chemical structure. This interpretation is consistent with previous reports indicating that bioactive compounds such as polyphenols do not chemically react with the oleogel matrix but are instead physically entrapped within the network structure [56].

The overall spectral similarity between control and colorant-enriched oleogels further supports the hypothesis that natural pigments are incorporated into the system without disrupting the triglyceride structure. Accordingly, the oleogel matrix can be considered a physically stabilized lipid network capable of encapsulating functional compounds while maintaining chemical integrity, which is particularly advantageous for food applications requiring stability without chemical modification of the lipid phase [22,56]. A study investigating the effect of β-carotene incorporation into oleogels reported similar findings, indicating that no peak shifts or the appearance of new peaks were observed in the β-carotene-containing oleogels. The authors suggested that these results indicate that β-carotene interacts with the other oleogel components only through physical interactions [29]. Another study reporting similar findings [57] indicated that no characteristic peaks corresponding to β-carotene or resveratrol were detected in the FTIR spectra. The authors concluded that the incorporation of β-carotene and resveratrol into oleogels contributed to oleogel formation predominantly through physical interactions rather than chemical interactions.

In the food industry, methods for solidifying vegetable oils are frequently used to meet the need for solid fats in specific products. Some of these methods, such as hydrogenation, can pose application challenges, leading to the formation of different covalent bonds in the oil and changes in its triglyceride structure. The fact that the products we produced in our study do not contain these negative aspects demonstrates that they can be a suitable alternative for use in the food industry for this purpose.

COOKIE

Dough Color Properties

**Figure 13:**
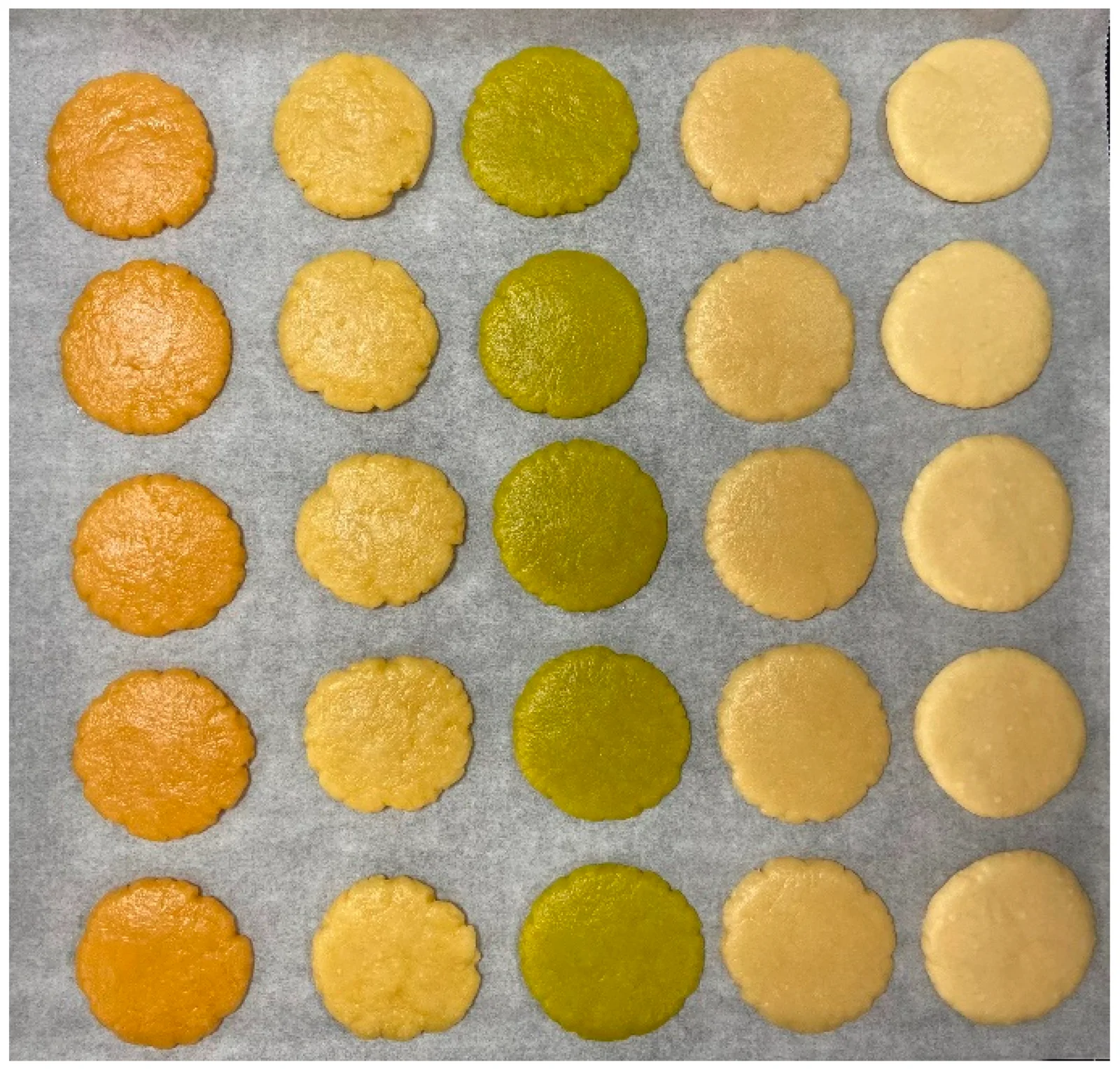
Appearance of cookie dough samples (before baking). From left to right: cookies prepared with RO, OO, GO, CO, and margarine, respectively.

Naturally colored oleogels were used as an alternative to solid fat in the cookie sample, which requires solid fat for production. Five different types of cookies were produced: a control sample using margarine as solid fat, and cookies using RO, OO, GO, and CO. The appearance of the produced cookies is shown in Figure 13.

The highest L* value was observed in the margarine dough, whereas the lowest value was recorded in the GO dough (Table 5). In terms of a* values, the highest result was obtained in RO dough (6.28) and the lowest result in GO dough (−2.13), which was significantly different from the other dough samples (*p* < 0.05). No statistically significant difference was observed in terms of a* value among the remaining dough formulations. The same observation was made when comparing the colors of the oleogels, with RO having the highest and GO the lowest a* values (Figure 6). It was suggested that the high a* value of RO may be due to the use of natural tomato color pigments found in its production. The fact that cookies prepared with RO and GO had the highest and lowest a* values among the samples shows that oleogels can retain their colors even within the cookie dough. Regarding b*values, the highest value was found in the OO dough with a statistically significant difference, while the lowest value was recorded in the CO dough. The fact that the b* value is highest in OO indicates that the color of the oleogel is reflected in the dough, meaning that the beta-carotene from carrots contributes to the color even in the cookie dough.

Overall, the results show that different types of fat and oleogel significantly affect the color characteristics of the cookie dough. Margarine increased the lightness (L*) of the dough, resulting in a brighter appearance, while spinach-based oleogel created a darker coloration. RO increased redness (a*), leading to more intense red tones, GO reduced redness, resulting in green tones, and OO increased yellowness (b*), resulting in more pronounced yellow tones. Accordingly, the color pigments of tomatoes, spinach, and carrots used in maceration played an effective role in the color of the cookie dough.

It was observed that the produced cookie doughs had lighter colors compared to the oleogels from which they were prepared. This could be attributed to the influence of other ingredients used in cookie making, such as flour and baking powder. These ingredients may have diluted or masked the oleogel’s color contribution, resulting in a weaker expression of the oleogel’s original color in the final cookie doughs.

Previous studies have reported lower L*, a*, and b* values in cookie doughs prepared with oleogels compared to those formulated with conventional solid fats [58]. However, due to the inherent color contributions of the oils used in the present study, a direct comparison with the literature is limited. Nevertheless, the differences presented demonstrate that the type of oleogel used significantly affects the color properties of the cookie dough systems.

Although all dough samples exhibited perceptible color differences relative to the control no statistically significant differences were observed among the dough samples (*p* > 0.05) (Table 5). Similar findings have been reported for oleogel-based dough systems, where ΔE* values ranging from 2.84 to 10.57 were observed among doughs formulated with different oleogelators, indicating that the incorporation of different oleogels can alter dough color relative to the reference formulation [50]. However, the relatively narrow range of ΔE* values observed in the present study suggests that the different colorants resulted in comparable overall color differences in the dough matrix. The color differences observed are associated with changes in the L*, a*, and b* coordinates caused by the incorporation of the colored oleogels into the dough formulation.

The appearance and color of cookies are strongly influenced by the type of fat used in their formulation and its compositional characteristics. The fatty phase may contain pigments and other minor components that can contribute to the color of the resulting cookies. In addition, cookie color is substantially affected by non-enzymatic browning reactions, particularly Maillard reactions, which occur during baking. The extent and intensity of these reactions are influenced by several factors, including baking temperature, pH, moisture content, and the composition of the ingredients. Therefore, differences in the type and composition of the fat used, as well as the presence of colorants and other minor compounds, may contribute to variations in the color characteristics of cookies [59]. All these properties are the main reasons for the color differences in the cookies. The effect of baking on color difference is particularly evident from the ΔE value between dough and cookie samples.

The ΔE* values of the cookie samples ranged from 5.97 to 12.67 (Table 5). All samples exhibited ΔE* values above 3, indicating that the color differences were perceptible to the human eye. These results are consistent with previous studies on oleogel-based cookies. In a study on cookies prepared with hemp oil-based oleogels, ΔE* values ranging from 5.96 to 15.55 were reported, demonstrating considerable color differences among cookie formulations [11]. Similarly, ΔE* values of 4.03 and 5.74 were reported for cookies formulated with oleogel-based Pickering emulsions, with the observed color differences being associated with the type of fat or oleogel used [60]. Therefore, the differences observed among the cookie samples in the present study may be attributed to variations in the color characteristics of the oleogels and the colorants incorporated into the formulations, as well as changes occurring during baking.

The ΔE* values between the unbaked and baked cookies ranged from 5.32 to 24.52. The highest color difference (24.52) was observed in the cookie containing chlorophyll, indicating a pronounced change in color during baking. This substantial change may be associated with the thermal instability of chlorophyll pigments. Chlorophylls are susceptible to heat-induced degradation and can be converted into pheophytin derivatives during thermal processing, resulting in marked changes in the green color and overall color characteristics of food products. Previous studies have demonstrated that heat treatment causes chlorophyll degradation accompanied by changes in instrumental color parameters and surface color [61]. Therefore, the higher ΔE* observed for the chlorophyll-containing cookie may be attributed, at least in part, to the degradation and transformation of chlorophyll pigments during baking. In addition, the formation of brown pigments through non-enzymatic browning reactions during baking may have further contributed to the overall color change [59].

Dough Texture Properties

Regarding springiness, the RO dough showed the highest value and differed significantly from all other dough samples (*p* < 0.05) (Table 6). The high springiness of RO dough indicates a more elastic structure. It is thought that the color pigment in RO may have contributed to the dough gaining a more elastic structure by supporting the formation of a crystalline network in the oleogel structure. The springiness values obtained in the present study were consistent with previous reports on oleogel-based cookie systems, including studies reporting values between 0.510 and 0.583 mm [13] and 0.99–1.00 mm [11]. In addition, a study using cookies prepared with 2.5% candelilla wax-based oleogels reported a springiness value of 0.48 ± 0.04 mm [58], which is consistent with the findings of the present work.

In terms of hardness, no statistically significant differences were observed among the oleogel-based dough samples, whereas the lowest hardness value was recorded in the margarine-based dough. The hardness values obtained in this study were highly consistent with those reported by [11], indicating comparable textural behavior across similar formulations.

Physical Properties of Cookies

The dimensions of cookie samples were measured to evaluate the effect of oleogels on spreading behavior (Table 7). According to the physical analysis results, the lowest transverse spread value was observed in the margarine-based cookies; however, no statistically significant differences were found among oleogel-based cookie samples for this parameter (*p* < 0.05).

**Figure 14:**
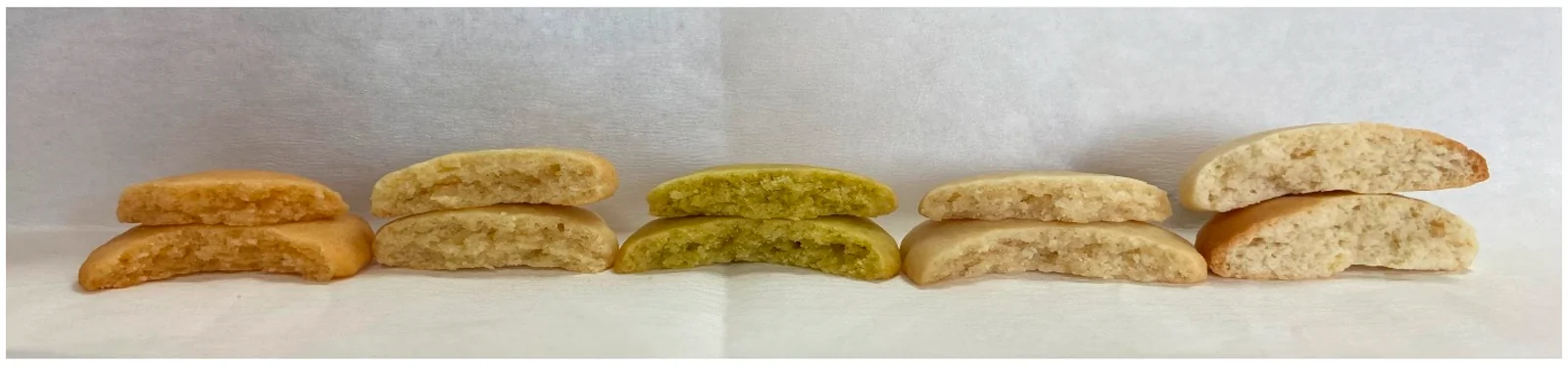
Fracture surface images of the cookie samples. From left to right: cookies prepared with RO, OO, GO, CO, and margarine, respectively.

Regarding longitudinal expansion (baking rise), the highest value was obtained in the margarine-based cookies, which differed significantly from all other formulations (Figure 14). Additionally, the margarine-based cookies exhibited approximately 1.5 times the rise compared to oleogel-based cookies (Table 7). In a study using margarine as a control, cookies prepared with different wax-based oleogels showed similar trends, where the thickness of margarine-based cookies was significantly higher than that of oleogel-based formulations, consistent with the present findings [12].

Oleogel-based cookies exhibited significantly higher spread ratios than the margarine control, whereas no significant differences were observed among the oleogel formulations (Table 7). An increase in spread ratio is generally associated with reduced aeration in cookie dough systems. In the present study, oleogel-based cookies exhibited higher spread ratios compared to margarine-based cookies. Similarly, previous studies have reported higher spread ratios in cookies prepared with sunflower oil compared to those formulated with margarine and hydrogenated fats [12], which may explain the higher spread behavior observed in oleogel-based formulations in the present work. Another study [62] also reported that cookies prepared with oleogels exhibited increased diameter and reduced height, this was considered an indicator of the desired spreading property and is highly consistent with the results of our study. Consistent with our findings, previous studies [62] have demonstrated that the use of shortening in cookie production significantly decreases the spread ratio.

The dimensional characteristics of the cookies in the present study were strongly influenced by the type of fat used. The oleogel-based cookies exhibited a greater diameter and lower height than the margarine-containing control, resulting in significantly higher spread ratios. This finding is consistent with the results reported in which cookies prepared with candelilla wax- and beeswax-based oleogels showed greater diameters and lower heights than the shortening control [63]. In that study, the spread factor increased from 8.06 for shortening-based cookies to 9.20 and 9.55 for candelilla wax- and beeswax-based oleogel cookies, respectively [63]. The authors attributed the increased spreading to the different structural and thermal properties of oleogels and also reported that an increase in spread factor was associated with reduced aeration of the cookie dough. Similarly, the higher spread ratio observed in the present oleogel-based cookies may indicate greater lateral dough flow during baking, accompanied by a reduction in vertical expansion. The lower height of the oleogel cookies may therefore be related to the greater mobility of the oleogel-derived oil phase during heating, which facilitates dough flow and promotes expansion in the horizontal direction. Overall, the agreement between the present findings and those reported in the literature suggests that replacing conventional structured fats with oleogels can substantially modify the dimensional properties of cookies by promoting lateral spreading at the expense of vertical development.

The expansion ratio of the cookies was significantly affected by the type of fat used (*p* < 0.05). This result is consistent with the greater diameter and lower height observed in the oleogel-based cookies, suggesting that oleogel incorporation promoted lateral spreading rather than vertical expansion during baking. In a previous study, oleogel replacement was reported to alter the dimensional properties of cookies, resulting in greater diameter and lower height compared with shortening, with higher spread factors associated with lower aeration of the dough [63]. Similarly, demonstrated that the expansion ratio of biscuits was strongly dependent on the type of fat and oleogelator used, with values ranging from approximately 193% to 338%, confirming that the structure and thermal behavior of the fat phase can substantially influence baking expansion [64]. Therefore, the lower expansion ratios observed in the present oleogel formulations may be attributed to differences in fat structure and melting behavior, which favored horizontal spreading of the dough and limited its vertical expansion during baking.

During baking, the melting of oleogels may increase the amount of free oil in the dough matrix, leading to a softer structure. This free oil reduces internal dough rigidity, thereby promoting spreading behavior. As a result, cookie diameter increases while thickness decreases [65], which is consistent with the findings of the present study. In terms of weight loss during baking, no statistically significant differences were observed among cookie samples, indicating that the type of fat system did not substantially affect moisture loss.

Cookie Color

**Figure 15:**
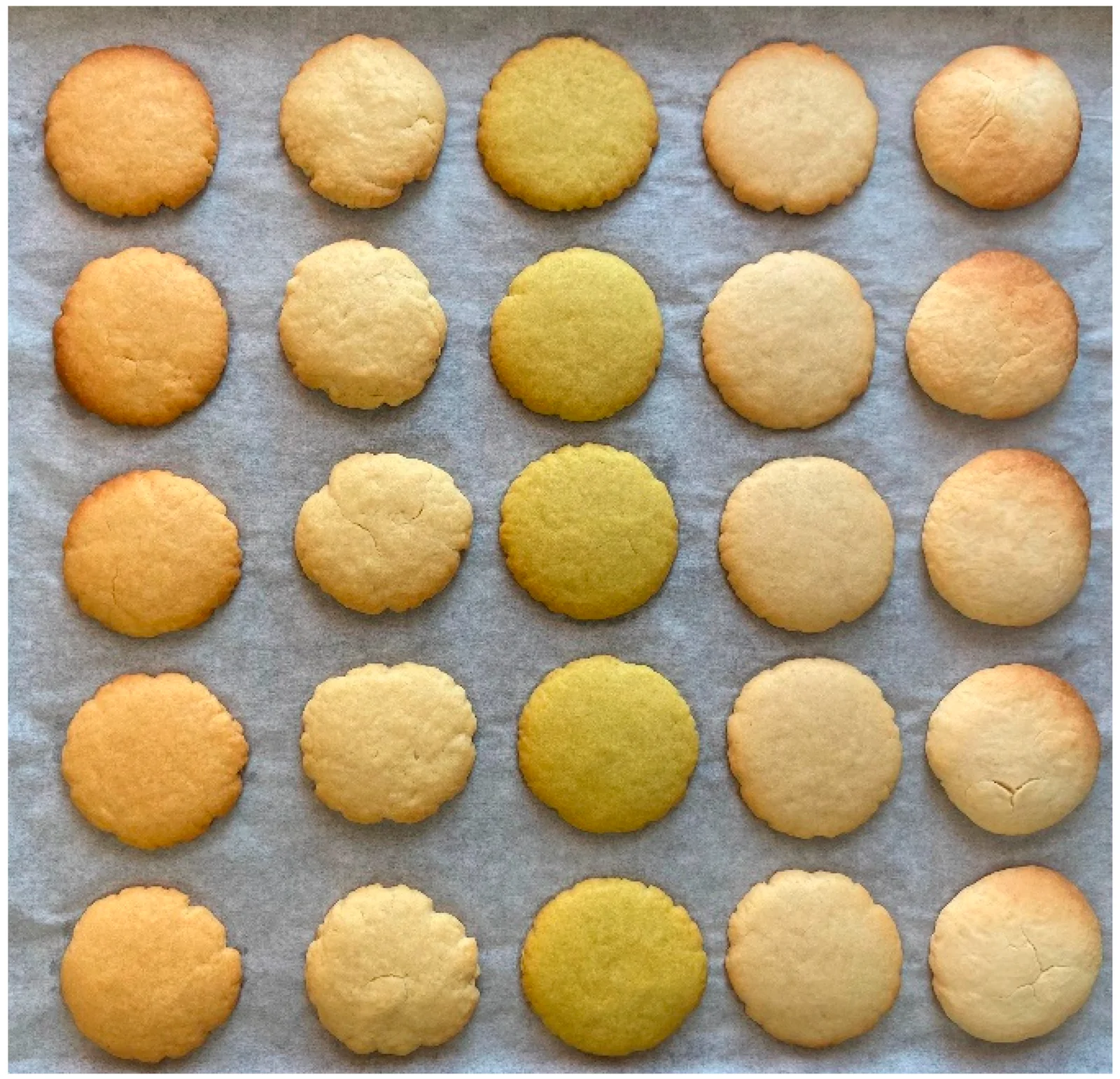
Appearance of cookie samples (after baking). Baked cookie samples from left to right: cookies prepared with RO, OO, GO, CO, and margarine, respectively.

In the present study, identical formulations and baking conditions were applied during cookie preparation; therefore, observed color differences can be attributed solely to the inherent color characteristics of margarine and oleogel systems (Figure 15).

**Figure 16:**
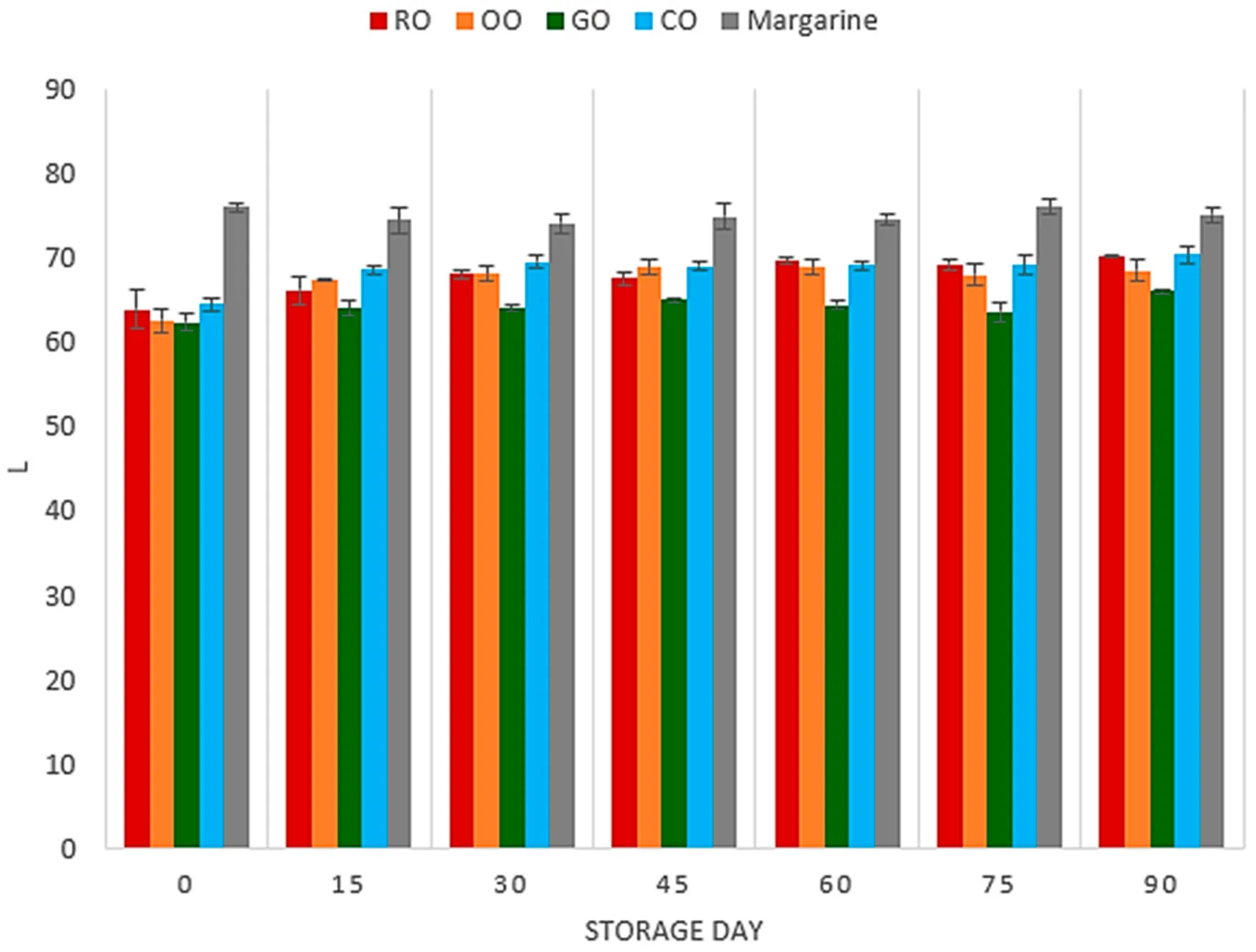
Changes in L values of cookie samples during 90 days of storage at room temperature. CO: Control Oleogel (without color), RO: Red Oleogel prepared with tomato-derived color pigment-enriched oil), OO: Orange Oleogel (prepared with carrot-derived color pigment-enriched oil), GO: Green Oleogel (prepared with spinach-derived color pigment-enriched oil).

Overall, no statistically significant differences in L* values were detected among samples during storage (*p* < 0.05). However, when evaluated at individual storage time points (0, 15, 30, 45, 60, 75, and 90 days), the highest L* values were consistently recorded in margarine-based cookies, which differed significantly from all oleogel-based formulations (*p* < 0.05). Among oleogel samples, no significant differences were generally observed; nevertheless, spinach-based oleogel cookies exhibited the lowest L* values from day 30 onward and were significantly different from the other formulations (*p* < 0.05). The observed increases in L* values in some oleogel-based samples during storage may be associated with oxidative changes in pigments and/or surface lightening due to moisture loss over time. Both oleogel type and storage duration influenced the color stability of cookies, while margarine-based formulations showed more stable lightness throughout storage (Figure 16).

Consistent with these findings, a study using margarine as a control reported that margarine-based cookies had a higher L* value (68.31) than oleogel-based cookies (53.63–63.36) [11]. Similarly, another study reported higher L* values in margarine-based cookies than in oleogel-based formulations and found no significant changes in L* values during 60 days of storage [14]. It has also been reported that cookies prepared with oleogels generally exhibit lower brightness than those prepared with margarine [58], which is in agreement with the results of the present study.

The a* values indicated that the cookie prepared with RO exhibited a more pronounced red color compared with the other samples. Cookies prepared with GO showed negative values, indicating greenness. When comparing the colors of the oleogels (Figure 6), it is observed that the a* values of RO and OO are higher than the a* value of CO. Although RO showed a decrease after baking, it still had a higher a* value than CO, but the a* value of OO remained the same as CO. This reveals that the red color given to the cookies by the natural color pigments extracted from tomatoes and carrots is affected by the baking temperature (Figure 14).

**Figure 17:**
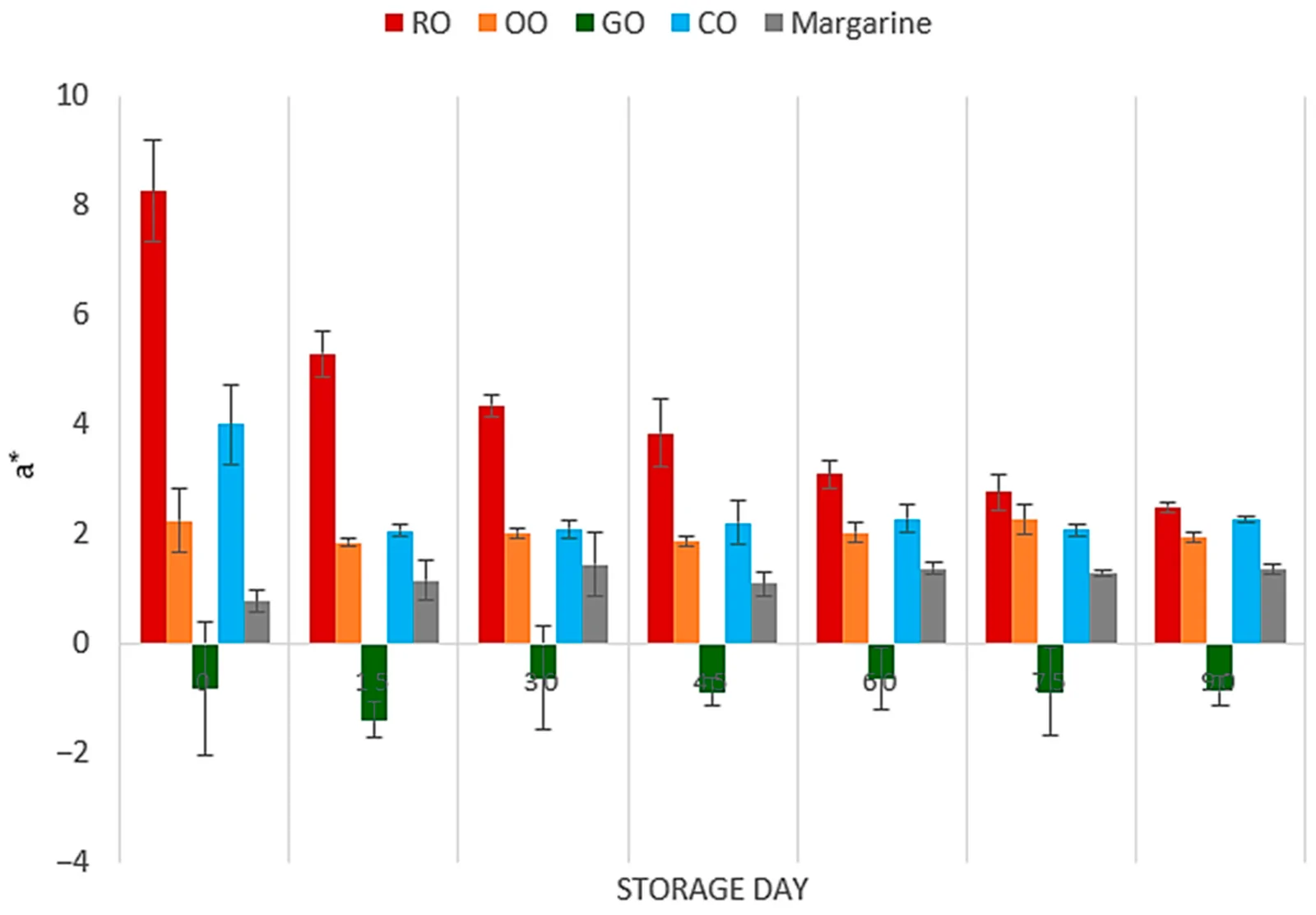
Changes in the a* values of cookie samples during 90 days of storage at room temperature. CO: Control Oleogel (without color), RO: Red Oleogel (prepared with tomato-derived color pigment-enriched oil), OO: Orange Oleogel (prepared with carrot-derived color pigment-enriched oil), GO: Green Oleogel (prepared with spinach-derived color pigment-enriched oil).

Changes in the a* values of cookies during storage indicated a time-dependent decrease in RO cookies. In particular, a* statistically significant reduction was observed between days 0 and 15 (*p* < 0.05). In contrast, no significant changes in a* values were detected throughout storage in OO, GO, CO, and margarine-based cookies (*p* < 0.05). Among all samples, the lowest a* values were consistently observed in margarine-based cookies throughout the storage period (Figure 17).

When evaluated at individual storage time points, RO cookies exhibited significantly higher a* values than all other formulations at days 0, 15, 30, 45, and 60. During these periods, no significant differences were observed among the remaining samples. However, at days 75 and 90, RO cookies no longer showed statistically significant differences compared to the other formulations, indicating a convergence in redness values over extended storage. This behavior may be associated with the oxidative degradation of tomato-derived pigments and color losses induced by storage conditions.

Consistent with the present findings, a previous study reported that increasing the proportion of oleogel used instead of margarine led to higher a* and b* values in cookie formulations [14], supporting the influence of oleogel incorporation on color attributes. Another noteworthy result of this study is that when comparing the color of the dough and the baked cookie (initial analysis), an increase in the a* value was recorded in all samples. This indicates increased redness in the samples. This result can be attributed to Maillard reactions, especially caramelization, during baking, due to the sugar content of the products.

**Figure 18:**
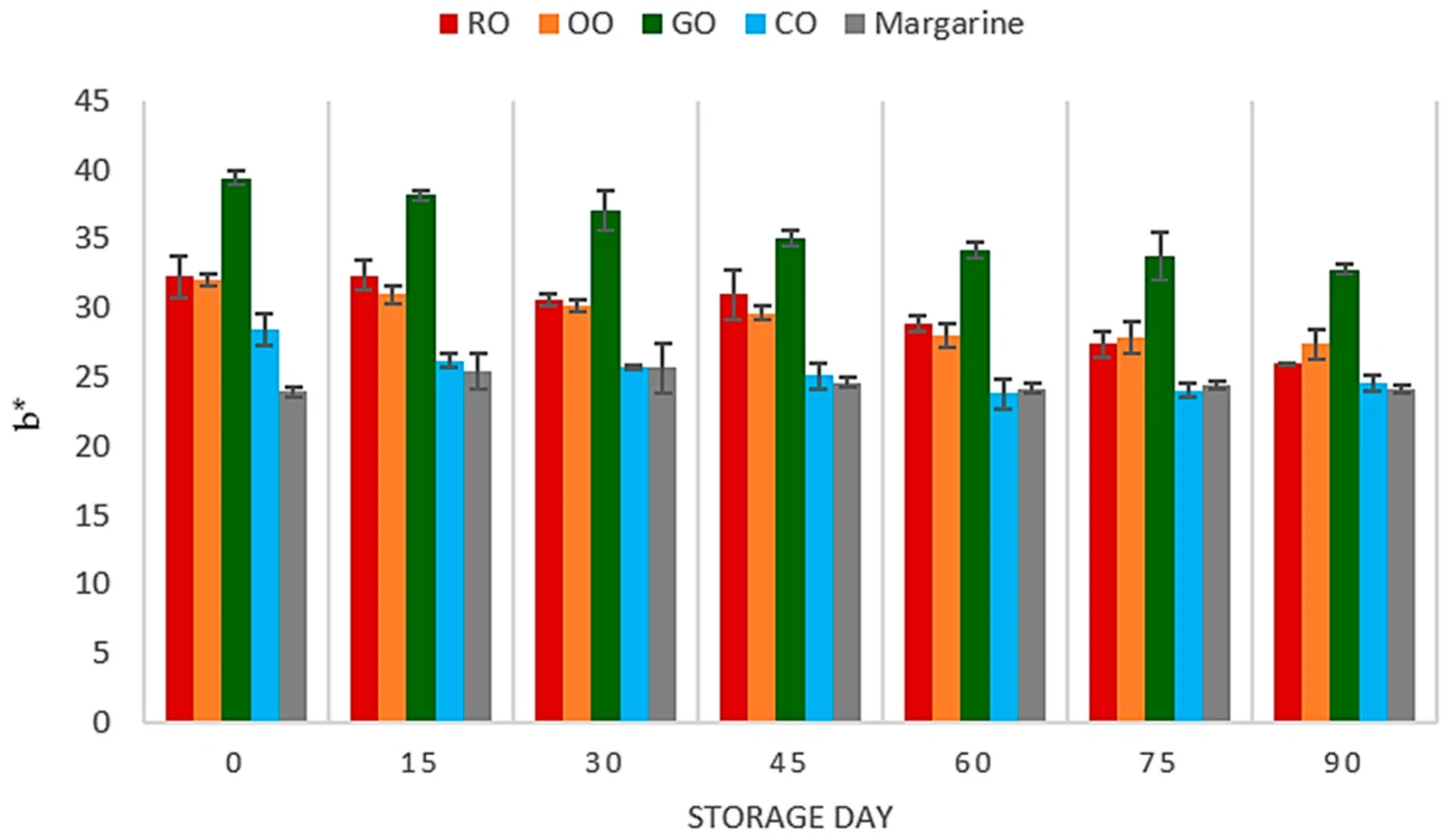
Changes in b* values of cookie samples during 90 days of storage at room temperature. CO: Control Oleogel (without color), RO: Red Oleogel (prepared with tomato-derived color pigment-enriched oil), OO: Orange Oleogel (prepared with carrot-derived color pigment-enriched oil), GO: Green Oleogel (prepared with spinach-derived color pigment-enriched oil).

The highest b value was recorded in RO cookies, while the lowest was in margarine cookies (Figure 18). RO and OO showed very close values, while CO cookies exhibited a higher b value than margarine cookies.

Changes in b* values during storage indicated a general decreasing trend in RO, OO, GO, and CO-based cookies. However, no statistically significant differences were observed between consecutive storage time points. In contrast, margarine-based cookies showed no significant change in b* values throughout the storage period, indicating greater stability in yellowness.

When evaluated across storage days, GO cookies consistently exhibited the highest b* values throughout the entire storage period and were significantly different from all other formulations at each time point. RO and OO cookies showed similar b* values across all storage periods, with no statistically significant differences observed between these two formulations.

The decreasing trend observed in RO, OO, and CO cookies over time may be associated with oxidative transformations of pigments and color degradation induced by storage conditions. In contrast, the relatively stable b* values observed in margarine-based cookies suggest a higher resistance to color changes in this formulation.

Supporting the present findings, a previous study reported that margarine-based control cookies exhibited lower b* values than oleogel-based cookies, with values around 22.65, which is comparable to those obtained in the present study [2]. Similarly, another study reported b* values of 28.97 for margarine-based cookies, which were lower than those for oleogel-based cookies (32.91–33.55) c, in agreement with the trends observed in the current work.

Cookie Hardness

Hardness is a key quality attribute in cookies, as excessively hard products are generally perceived as lower in quality, which can negatively affect consumer acceptance and purchase intention [2]. Hardness is defined as the resistance of a material to deformation under an applied force.

**Figure 19:**
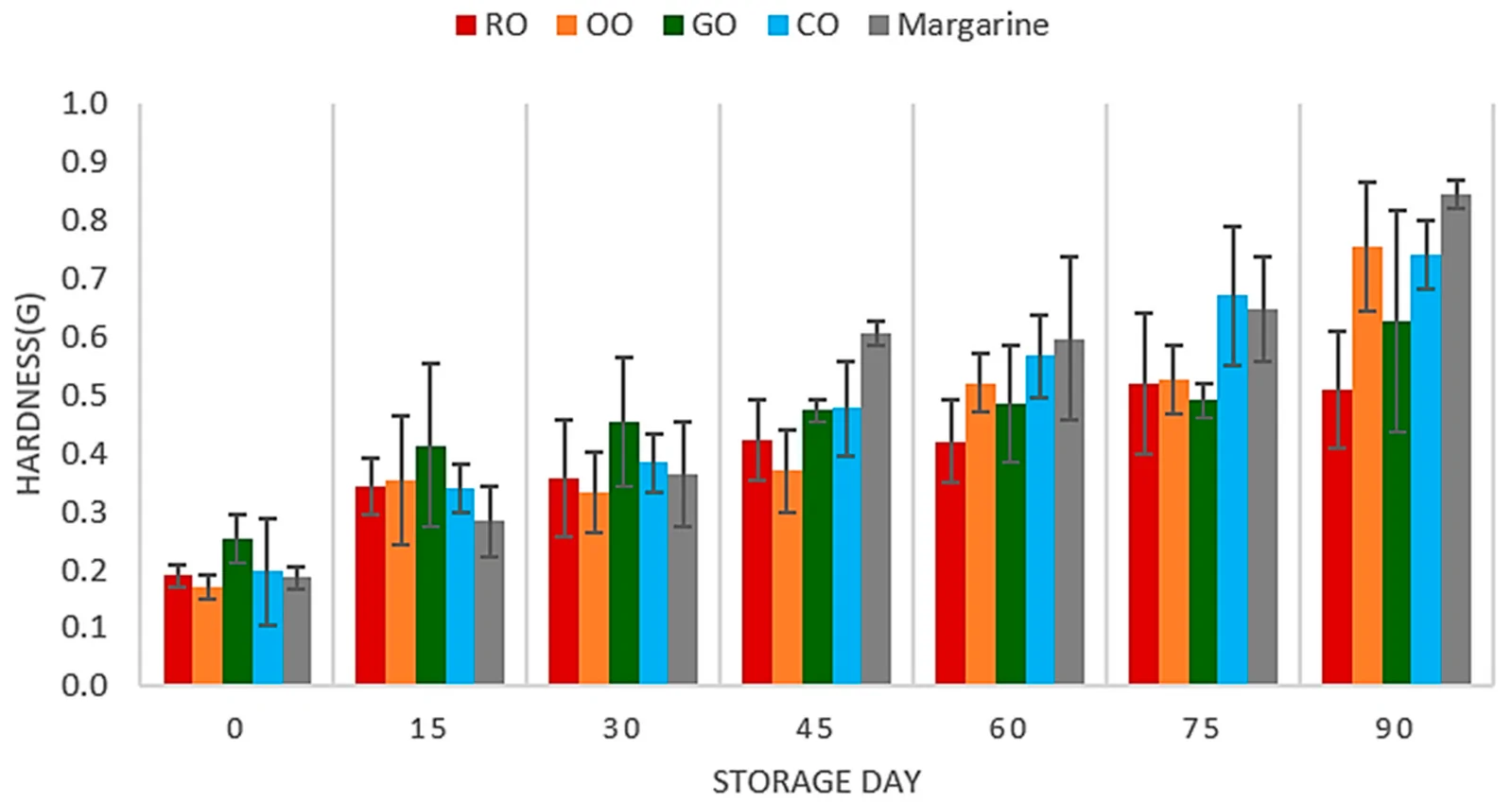
Changes in hardness values of cookie samples during 90 days of storage at room temperature. CO: Control Oleogel (without color), RO: Red Oleogel (prepared with tomato-derived color pigment-enriched oil), OO: Orange Oleogel (prepared with carrot-derived color pigment-enriched oil), GO: Green Oleogel (prepared with spinach-derived color pigment-enriched oil).

There was no significant difference in the hardness values of the cookies on the first day of analysis (*p* < 0.05). No statistically significant differences were observed between consecutive storage days for RO, OO, CO, and margarine-based cookies (*p* < 0.05). The hardness values of the margarine cookies showed different increases than those of the oleogel cookies, and the highest hardness values were recorded in the margarine cookies after the 30th day (Figure 19). However, a general increasing trend in hardness values was observed throughout the storage period. This increase may be associated with physical changes occurring during storage, such as moisture loss, structural densification, and starch retrogradation.

Consistent with the present findings, previous studies have reported that cookies formulated with oleogels generally exhibit lower hardness values compared to those prepared with margarine [2,4,12,14,64] supporting the influence of fat replacement on cookie texture and overall product quality.

## 4. Conclusions

This study demonstrated that sunflower oil-based oleogels structured with candelilla wax and enriched with natural pigments from tomato, carrot, and spinach can be successfully used and applied in cookie formulations as an alternative to conventional solid fats. The results confirmed that oleogelation effectively immobilizes liquid oil through a physically structured network without altering its chemical composition, while maintaining high oil-binding capacity and acceptable gelation properties.

Although oxidative parameters (peroxide, free fatty acid, and p-anisidine values) increased during storage, all samples generally remained within acceptable quality limits, indicating that oleogel systems can provide adequate oxidative stability. FTIR analysis further confirmed the absence of chemical interactions between bioactive compounds and the lipid matrix, supporting a physically stabilized system.

Color measurements revealed that natural pigments significantly affect the product’s appearance, and spinach-based oleogel (GO) exhibited superior color stability during storage despite chlorophyll’s sensitivity. In this study, characteristic red, orange, and green colors from tomatoes, carrots, and spinach were incorporated into the oil, and these colors were evident from the initial maceration process to the final product, the cookies. In cookie applications, oleogel-based formulations produced softer and more spreadable doughs with lower hardness compared to margarine-based controls, although baking weight loss remained unchanged.

Overall, oleogels represent a promising fat-replacement strategy for bakery products, offering structural functionality, acceptable stability, and the added advantage of natural color incorporation. These findings support their potential for use in developing healthier, more functional cookie formulations.

Although the present study demonstrates the potential of oleogels as structured lipid systems, further research is needed to confirm their suitability for broader industrial applications. Future studies should investigate their physicochemical, oxidative, and storage stability under different processing and storage conditions, as well as their functional performance in different food systems. Such investigations would provide a better understanding of the stability and functionality of oleogels and help establish their potential for wider industrial use as alternatives to conventional solid fats.

## Figures and Tables

**Table 1:** Oil-binding capacity (%), gelation time (minutes), and melting point (°C) of oleogel samples.

	RO	OO	GO	CO
Oil-binding Capacity	99.56 ± 0.06 a	99.84 ± 0.09 a	99.44 ± 0.23 a	99.78 ± 0.05 a
Gelation Time	10.67 ± 0.55 a	10.61 ± 0.38 a	10.93 ± 0.29 a	10.62 ± 0.83 a
Melting point	71.88 ± 2.81 a	71.80 ± 2.90 a	72.81 ± 2.70 a	74.75 ± 0.86 a

Values are expressed as mean ± standard deviation. Different letters indicate significant differences (*p* < 0.05). CO: Control Oleogel (without color), RO: Red Oleogel (prepared with tomato-derived color pigment-enriched oil), OO: Orange Oleogel (prepared with carrot-derived color pigment-enriched oil), GO: Green Oleogel (prepared with spinach-derived color pigment-enriched oil).

**Table 2:** Color differences (ΔE*) among oil samples.

	ROil	OOil	GOil	COil
ΔE* vs. COil	76.45 ± 2.69 a	61.24 ± 2.55 b	44.10 ± 3.11 c	-

Values are expressed as mean ± standard deviation. Different letters indicate significant differences (*p* < 0.05). COil: Control oil (without colorant sunflower oil), ROil: Oil obtained by extracting tomato pigments, OOil: Oil obtained by extracting carrot pigments, GOil: Oil obtained by extracting spinach pigments.

**Table 3:** Color differences (ΔE*) among oleogel samples.

	RO	OO	GO	CO
ΔE* vs. CO	35.23 ± 1.26 a	25.81 ± 3.11 b	15.68 ± 0.12 c	-

Values are expressed as mean ± standard deviation. Different letters indicate significant differences (*p* < 0.05). CO: Control Oleogel (without color), RO: Red Oleogel (prepared with tomato-derived color pigment-enriched oil), OO: Orange Oleogel (prepared with carrot-derived color pigment-enriched oil), GO: Green Oleogel (prepared with spinach-derived color pigment-enriched oil).

**Table 4:** Color differences (ΔE*) between oleogel and oil samples.

	RO-ROil	OO-OOil	GO-GOil	CO-COil
ΔE*	42.06 ± 1.76 a	38.99 ± 4.57 a	23.36 ± 3.52 b	35.83 ± 0.52 a

Values are expressed as mean ± standard deviation. Different letters indicate significant differences (*p* < 0.05). CO: Control Oleogel (without color), RO: Red Oleogel (prepared with tomato-derived color pigment-enriched oil), OO: Orange Oleogel (prepared with carrot-derived color pigment-enriched oil), GO: Green Oleogel (prepared with spinach-derived color pigment-enriched oil), COil: Control oil (without colorant sunflower oil), ROil: Oil obtained by extracting tomato pigments, OOil: Oil obtained by extracting carrot pigments, GOil: Oil obtained by extracting spinach pigments.

**Table 5:** Color parameters (L*, a*, b*) of cookie dough samples and the corresponding color differences (ΔE*).

	L*	a*	b*	ΔE* (Among Dough)	ΔE* (Dough-Cookie)	ΔE* (Among Cookie)
RO dough	54.89 ± 1.98 b	6.27 ± 0.41 a	27.28 ± 0.45 b	13.29 ± 4.63 a	10.14 ± 1.55 bc	7.50 ± 1.69 b
OO Dough	57.27 ± 3.20 b	0.09 ± 0.20 b	30.54 ± 2.26 a	11.69 ± 0.25 a	5.32 ± 2.04 c	5.97 ± 3.14 b
GO dough	46.86 ± 3.81 c	−2.13 ± 0.86 c	21.02 ± 0.50 c	11.85 ± 1.43 a	24.52 ± 1.20 a	12.67 ± 0.54 a
CO dough	56.64 ± 0.77 b	0.14 ± 0.27 b	19.15 ± 0.80 c	-	12.36 ± 1.21 b	-
Margarine dough	68.36 ± 1.15 a	0.60 ± 0.57 b	27.98 ± 1.02 b	13.33 ± 2.61 a	10.20 ± 3.74 bc	12.56 ± 0.20 a

Values are expressed as mean ± standard deviation. Different letters indicate significant differences (*p* < 0.05). CO: Control Oleogel (without color), RO: Red Oleogel (prepared with tomato-derived color pigment-enriched oil), OO: Orange Oleogel (prepared with carrot-derived color pigment-enriched oil), GO: Green Oleogel (prepared with spinach-derived color pigment-enriched oil).

**Table 6:** Texture parameters of cookie dough samples.

	Springiness (mm)	Hardness (g)
RO dough	1.61 ± 0.35 a	0.18 ± 0.005 a
OO Dough	0.62 ± 0.20 bc	0.17 ± 0.005 ab
GO dough	0.63 ± 0.10 c	0.17 ± 0.00 ab
CO dough	0.69 ± 0.10 bc	0.18 ± 0.007 a
Margarine dough	0.80 ± 0.10 bc	0.16 ± 0.005 b

Values are expressed as mean ± standard deviation. Different letters indicate significant differences (*p* < 0.05). CO: Control Oleogel (without color), RO: Red Oleogel (prepared with tomato-derived color pigment-enriched oil), OO: Orange Oleogel (prepared with carrot-derived color pigment-enriched oil), GO: Green Oleogel (prepared with spinach-derived color pigment-enriched oil).

**Table 7:** Physical Properties of cookie and cookie dough samples.

	Spread Ratio	Longitudinal Expansion (%) (Baking Rise)	Baking Weight Loss (%)
RO dough	4.71 ± 0.35 a	162.50 ± 12.50 b	11.68 ± 0.13 a
OO Dough	5.30 ± 0.05 a	137.50 ± 12.50 c	11.20 ± 0.49 a
GO dough	5.38 ± 0.35 a	137.50 ± 0.00 c	11.47 ± 1.32 a
CO dough	5.18 ± 0.56 a	137.50 ± 12.50 c	11.60 ± 0.85 a
Margarine dough	3.04 ± 0.04 b	212.50 ± 0.00 a	11.52 ± 0.25 a

Values are expressed as mean ± standard deviation. Different letters indicate significant differences (*p* < 0.05). CO: Control Oleogel (without color), RO: Red Oleogel (prepared with tomato-derived color pigment-enriched oil), OO: Orange Oleogel (prepared with carrot-derived color pigment-enriched oil), GO: Green Oleogel (prepared with spinach-derived color pigment-enriched oil).

## Data Availability

The original contributions presented in this study are included in the article. Further inquiries can be directed to the corresponding author.

## Abbreviations

The following abbreviations are used in this manuscript:

CDW	Candelilla wax
Coil	Control oil (Without colorant sunflower oil)
ROil	Oil obtained by extracting tomato pigments
OOil	Oil obtained by extracting carrot pigments
GOil	Oil obtained by extracting spinach pigments
CO	Control Oleogel (Without color)
RO	Red Oleogel (prepared with tomato-derived color pigment-enriched oil)
OO	Orange Oleogel (prepared with carrot-derived color pigment-enriched oil)
GO	Green Oleogel (prepared with spinach-derived color pigment-enriched oil)
OBC	Oil-binding capacity
PV	Peroxide value
p_AV	Para anisidine value
FTIR	Fourier Transform Infrared
FFA	Free Fatty Acid
